# The Solution Behavior of Dopamine in the Presence of Mono and Divalent Cations: A Thermodynamic Investigation in Different Experimental Conditions

**DOI:** 10.3390/biom11091312

**Published:** 2021-09-05

**Authors:** Antonio Gigliuto, Rosalia Maria Cigala, Anna Irto, Maria Rosa Felice, Alberto Pettignano, Demetrio Milea, Stefano Materazzi, Concetta De Stefano, Francesco Crea

**Affiliations:** 1 Dipartimento di Scienze Chimiche, Biologiche, Farmaceutiche ed Ambientali, Università degli Studi di Messina, V.le F. Stagno d’Alcontres, 31, I-98166 Messina, Italy; agigliuto@unime.it (A.G.); rmcigala@unime.it (R.M.C.); airto@unime.it (A.I.); mrfelice@unime.it (M.R.F.); dmilea@unime.it (D.M.); cdestefano@unime.it (C.D.S.); 2 Dipartimento di Fisica e Chimica, Università degli Studi di Palermo, V.le delle Scienze, ed. 17, I-90128 Palermo, Italy; alberto.pettignano@unipa.it; 3 Dipartimento di Chimica, Università “La Sapienza” di Roma, Piazzale A. Moro 5, I-00185 Rome, Italy; stefano.materazzi@uniroma1.it

**Keywords:** chemical speciation, metal complexes, catechol, sequestration, stability constants

## Abstract

The interactions of dopamine [2-(3,4-Dihydroxyphenyl)ethylamine, (Dop^−^)] with methylmercury(II) (CH_3_Hg^+^), magnesium(II), calcium(II), and tin(II) were studied in NaCl(aq) at different ionic strengths and temperatures. Different speciation models were obtained, mainly characterized by mononuclear species. Only for Sn^2+^ we observed the formation of binuclear complexes (M_2_L_2_ and M_2_LOH (charge omitted for simplicity); M = Sn^2+^, L = Dop^−^). For CH_3_Hg^+^, the speciation model reported the ternary MLCl (M = CH_3_Hg^+^) complex. The dependence on the ionic strength of complex formation constants was modeled by using both an extended Debye–Hückel equation that included the Van’t Hoff term for the calculation of enthalpy change values of the formation and the Specific Ion Interaction Theory (SIT). The results highlighted that, in general, the entropy is the driving force of the process. The sequestering ability of dopamine towards the investigated cations was evaluated using the calculation of pL_0.5_ parameter. The sequestering ability trend resulted to be: Sn^2+^ > CH_3_Hg^+^ > Ca^2+^ > Mg^2+^. For example, at *I* = 0.15 mol dm^−3^, *T* = 298.15 K and pH = 7.4, pL_0.5_ = 3.46, 2.63, 1.15, and 2.27 for Sn^2+^, CH_3_Hg^+^, Ca^2+^ and Mg^2+^ (pH = 9.5 for Mg^2+^), respectively. For the Ca^2+^/Dop^−^ system, the precipitates collected at the end of the potentiometric titrations were analyzed by thermogravimetry (TGA). The thermogravimetric calculations highlighted the formation of solid with stoichiometry dependent on the different metal:ligand ratios and concentrations of the starting solutions.

## 1. Introduction

Speciation is the process of formation of chemical species through a series of reactions (formation of complexes) that lead to the characterization of a component in a natural or artificial system. The more complicated the system where the interactions are investigated, the greater the degree of difficulty in fully defining the effective distribution of the chemical species. Therefore, a complete speciation model of a fluid can be formulated if all of the possible interactions among the components dissolved are considered. Each constituent of the natural and biological fluids assumes its own thermodynamic behavior, i.e., different speciation, as a function of different variables, such as temperature, ionic medium, ionic strength, component concentrations, and ligand:metal molar ratios [1,2,3]. Speciation studies, in the fields of clinical and environmental chemistry, are of great importance, since the polluting, toxic, or curative action of a given component depends on the specific species to which it originates in that natural fluid. In the context of biological and environmental monitoring, speciation involves three different levels of study: (1) species analysis of a specific element; (2) subdivision through specific chemical analysis of organic and inorganic species; (3) application to the analysis of information about the differences in the distribution of the different species of an element [1,2,3].

**Scheme 1:**
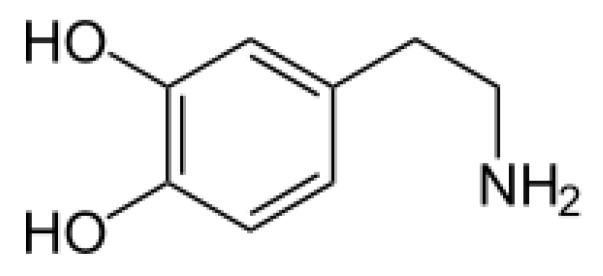
2-(3,4-Dihydroxyphenyl)ethylamine [dopamine].

Dopamine [2-(3,4-Dihydroxyphenyl)ethylamine (see Scheme 1)] is a neurohormone released by the hypothalamus. Its main function as a hormone is to inhibit the release of prolactin from the anterior pituitary lobe [4,5,6,7].

Dopamine is biosynthesized in the body (especially in the nervous tissue and adrenal medulla) by a mechanism that primarily involves the hydroxylation of the amino acid L-tyrosine (normally found in the diet) into L-DOPA through the enzyme tyrosine 3-monooxygenase, and subsequent decarboxylation of L-DOPA by the DOPA decarboxylase, which will remove the carboxyl group (-COOH) from the DOPA side chain.

In some neurons, dopamine is transformed into noradrenaline by dopamine beta-hydroxylase and after synthesis; it is here “packaged” into synaptic vesicles, which are then released into the synapses in response to a presynaptic action potential. The storage inside the vesicles has the purpose of protecting the molecule from degradation by the monoamine oxidase and is indispensable for the release of the neurotransmitter in the synaptic space by the nerve impulse.

As a medicinal, it is used in the treatment of neuropathology, such as Parkinson’s and schizophrenia. Specifically: Parkinson’s disease is associated with a decrease in the concentration of dopamine in the brain; therefore, it requires the use of drugs containing levodopa (LD) combined with a certain amount of carbidopa (CD), which makes LD available for transport to the brain and subsequently converted to dopamine in the basal ganglia. Schizophrenia is associated with an increase in dopamine concentration in the brain. In this case, the drug used blocks dopamine receptors in the brain [8].

Many years ago, this research group started a systematic investigation on the acid-base properties, solubility, and interaction of molecules of biological and pharmaceutical relevance towards different metal ions [9,10,11,12,13,14,15,16,17,18,19].

The acid-base properties of dopamine in NaCl aqueous solutions and the distribution between NaCl solutions/organic solvent at different experimental conditions (ionic strengths and temperatures) were already studied [10].

As a further contribution to the speciation studies of this molecule, the interactions towards different metal ions, such as CH_3_Hg^+^, Ca^2+^, Mg^2+^ and Sn^2+^, were investigated.

The studies were carried out by means of potentiometric titrations in NaCl(aq) in a wide range of component concentrations and ligand/metal molar ratios, at different ionic strengths and temperatures.

The dependence of the complex formation constants on the ionic strength was investigated by means of an extended Debye–Hückel equation that included a Van’t Hoff term for the calculation of the enthalpy change values of formation of the metal/dopamine complexes. The Specific Ion Interaction Theory (SIT) approach was used to calculate the specific ion interaction parameters [20,21,22].

The effective sequestering ability of dopamine towards the different metal ions here considered was quantified at selected ionic strengths, pHs, and temperatures, by using a widely tested approach [23,24].

For Ca^2+^/Dop^−^ system, some of the precipitates collected at the end of the titrations were analyzed by means of thermogravimetry (TGA).

## 2. Materials and Methods

### 2.1. Chemicals

Dopamine solutions were prepared by weighing the corresponding hydrochloric salt without further purification. The purity was checked potentiometrically by alkalimetric titrations and resulted to be >99.5%. Sodium chloride aqueous solutions were prepared at different ionic strengths from dilution of a concentrated solution obtained by weighing pure salt previously dried in an oven (Thermo Fisher Scientific, Waltham, MA, USA) at *T* = 383.15 K for 2 h. Sodium hydroxide and hydrochloric acid solutions were prepared from concentrated ampoules and standardized against potassium hydrogen phthalate and sodium carbonate, respectively.

Standard stock solutions of the metals (CH_3_Hg^+^; Ca^2+^; Mg^2+^, and Sn^2+^) were prepared from the corresponding chloride salts and were used without further purification. The standard stock solutions of SnCl_2_ were acidified with HCl to pH~2 and a piece of metallic tin was added to the solutions after the preparation, to prevent the oxidation of Sn^2+^ to Sn^4+^ and to hamper the formation of soluble and sparingly soluble hydrolytic species.

The concentration of the metal ions in the aqueous solutions were determined by means of complexometric titrations with ethylenediaminetetraacetic acid sodium salt (EDTA) [25].

All of the products were purchased from Sigma-Aldrich (Milano, Italy). All solutions were prepared with analytical grade water (ρ = 18 MΩ cm^−1^) using grade A glassware and preserved from atmospheric CO_2_ by means of soda lime traps.

Further details on the chemicals used for the investigation here carried out are reported in Table 1.

### 2.2. Apparatus and Procedure

#### 2.2.1. Potentiometric Titrations

The interactions of dopamine with the selected cations were studied potentiometrically by means of an apparatus consisting of an 809 model Metrohm Titrando system (Metrohm, Varese, Italy), connected to a half-cell Ross Type glass electrode (model 8101 from Thermo Fisher Scientific, Waltham, MA, USA), and coupled with a standard Ag/AgCl reference electrode. The system was connected to a personal computer and the parameters of the titrations were controlled by means of the Metrohm TiAMO 2.2 computer program (Metrohm, Varese, Italy), which allowed performing automatic titrations, by addition of desired amounts of titrant when the equilibrium state was reached and recording the e.m.f. of the solution under investigation. The estimated accuracy was ±0.15 mV and ±0.003 mL for e.m.f and titrant volume readings, respectively.

The measurements were carried out under continuous stirring and pure N_2_ flow in a thermostatted cell, connected to a Model D1-G-Haake thermocryostat (Gebrüder HAAKE GmbH, Karlsruhe, Germany), by means of water circulation in the outer chamber of the titration cell. Investigations were performed from *T* = 288.15 to 310.15 K; only for the CH_3_Hg^+^/Dop^−^ system, measurements were also carried out at *T* = 318.15 K.

Concerning the dependence of the stability constants on the ionic strength, interactions were studied at 0.15 ≤ *I*/mol dm^−3^ ≤ 1.0. For Ca^2+^ and Sn^2+^/Dop^−^ systems, at *T* = 288.15 and 310.15 K, only the ionic strength *I* = 0.15 mol dm^−3^ was investigated.

The titrated solutions consisted of different amounts of the cation, dopamine·HCl, an excess of hydrochloric acid and NaCl, for obtaining the desired ionic strength values. To investigate the possible formation of both mono- and/or polynuclear species, solutions were prepared in a wide range of cation to ligand molar ratios and were titrated with standard carbonate free NaOH, up to alkaline pH values, or to the formation of sparingly soluble species. Table 2 reports the experimental conditions employed in the investigations of the systems (components concentration, ligand:cation molar ratios).

For each titration, the total number of potentiometric experimental points collected varied between 60 and 100, in dependence on the possible formation of a sparingly soluble species. At least two titrations were performed for each experimental condition. Independent titrations of strong acid (HCl) solutions with NaOH solutions were carried out at the same experimental conditions of the systems investigated, with the aim of determining the electrode potential (E^0^) and the acidic junction potential (Ej = ja [H^+^]). In this way, the pH scale used was the free concentration scale, pH ≡ −log [H^+^], where [H^+^] is the free proton concentration (not activity). The reliability of the calibration in the alkaline range was checked by calculating the ionic product of water (pK_w_).

**Figure 1:**
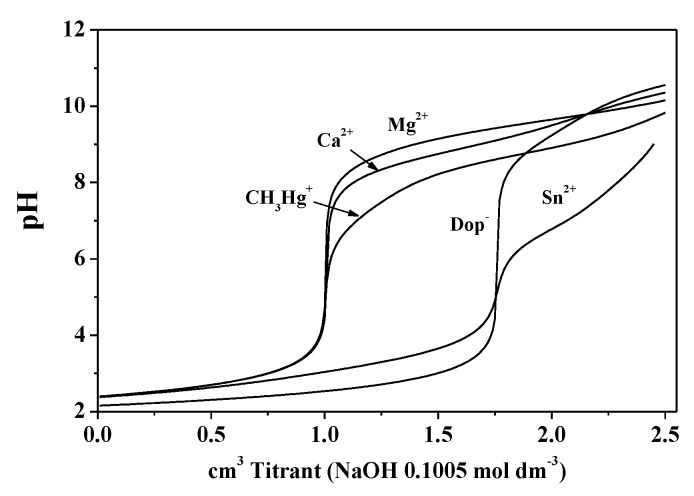
Titration curves of dopamine and of the different metal/Dop^−^ systems at *T* = 298.15 K and *I* = 0.15 mol dm^−3^. (Experimental conditions: *c*_Dop−_ = 3 mmol dm^−3^; *c*_Mn+_= 1.5 mol dm^−3^).

Figure 1 reports the titration curves of dopamine and of the metal/dopamine systems at *I* = 0.15 mol dm^−3^ and *T* = 298.15 K, where it is possible to observe quite different curve profiles, especially for the Sn^2+^ containing solution, due to the different acid-base properties of the metal ions and the strength of the interaction towards dopamine.

#### 2.2.2. Thermogravimetric Measurements

A thermo-analytic characterization was performed by a PerkinElmer TGA7 equipment (PerkinElmer, Milano, Italy). The investigated samples (approximately 2–10 mg) were heated in platinum crucibles in the temperature range 293–1123 K, under an atmosphere of air (gaseous mixture of nitrogen and oxygen with 80% and 20%, *v*/*v*, respectively) at a flow rate of 100 mL min^−1^ and a scanning rate of 10 K min^−1^. These conditions allowed the best possible resolution of the thermogravimetric curves.

### 2.3. Calculations

#### Computer Programs

All parameters of the potentiometric titrations (standard electrode potential (E^0^), liquid junction potential coefficient (j_a_), ionic product of the water (K_w_), analytical concentration of reagents and formation constants) were determined by using the non-linear least square minimization method and the BSTAC program (further details are reported in ref [26]), by solving the component mass balance equations to obtain explicit functions for the average number of ligand bound to the metal.

By using the BSTAC program, we minimized the error square sum in the electromotive force (E = e.m.f.)

U = ∑W·(E_exp_ − E_calcd_)^2^ (1)

Weights for each point of the titration curves are given as (s^2^ = variance)

W = 1/s^2^ (2)

s^2^ = sE^2^ + ((∂Ei)/(∂Vi))^2^·sV^2^ (3)

where sE^2^ and sV^2^ are the estimated variances for the e.m.f. and volume, respectively.

The program uses a Gauss–Newton technique for the refinement of the equilibrium constants:

s = (A^T^·W·A)^−1^·(A^T^·W)·e (4)

where, A = matrix of the partial derivatives ∂y/∂p (y is an independent variable and p the i^th^ point of the potentiometric measurement); W = weight matrix; e = residuals vector; s = shifts vector. Different independent variables, y, can be used for the refinement of the equilibrium constants from the potentiometric data, such as electromotive force (e.m.f.), v (titrant volume), X (analytical concentrations), n (average number of ligands bound to the metal), Z (average number of protons displaced per metal ion), etc.

The LInear And Nonlinear Analysis (LIANA) computer program, which minimizes the error square sum in y of an equation y = f(x_i_) (a generic function given by the user), by using the general Levenberg–Marquardt–Gauss–Newton Method, was employed for the determination of the equilibrium constants at infinite dilution, and the corresponding parameters for the dependence on the ionic strength, and of Specific Ion Interaction Theory parameter of the ion-pairs [26].

The ES4ECI [27] or Hyss [28] programs were employed for the calculation of the formation percentages of the species present in solution at the equilibrium and to draw both the distribution and the sequestration diagrams in different conditions. The HySS program allows considering the formation of the sparingly soluble species, by using the solubility product of the species in the speciation model.

Within the manuscript, if not differently specified, hydrolysis (q = 0, r < 0) constants of metal cations, protonation (p = 0) constants of the ligands (L^z−^), and complex formation constants are given according to the overall equilibrium:

logβ_pqr_: pM^n+^ + qL^z−^ + rH^+^ = M_p_L_q_H_r_^(np−zq+r)^ (5)

The errors associated to formation constants, enthalpy, and entropy change values and parameters for the dependence on ionic strength are expressed as ± standard deviation (Std. Dev.).

### 2.4. Dependence of the Stability Constants on the Ionic Strength and Temperature

The dependence of the formation constants on ionic strength was studied both by means of the Extended Debye–Hückel (EDH) equation and specific ion interaction theory (SIT) approach (general information can be found, e.g., in [20,21,22,29,30].

#### 2.4.1. Extended Debye–Hückel (EDH)

The dependence of the formation constants on the ionic strength and temperature was modeled by means of an extended Debye–Hückel (EDH) equation, allowing the calculation of the complex formation constants at infinite dilution and the enthalpy change of formation, when measurements were also carried out at different temperatures:

logβ_pqr_ = log^T^β_pqr_ − z*·A·√*I*/(1 + 1.5·√*I*) + C·*I* + L·(1/298.15 − 1/*T*)·52.23 (6)

where 52.23 is 1/(R·ln10) and C is the adjustable parameter for the dependence of formation constants on ionic strength in the molar concentration scale; this parameter can be expressed by the following equation: C = c_0_p* + c_1_z*; p* = ∑p_reactants_ − ∑p_products_ and z* = ∑z_reactants_^2^ − ∑z_products_^2^, where z and p are the charge and the stoichiometric coefficient, respectively. Log^T^β_pqr_ is the formation constant at infinite dilution and A is the Debye–Hückel term, expressed with respect to its dependence on the temperature, as reported in the Equation (7), where 0.856 and 0.00385 are empirical parameters, *T* = 298.15 K is the reference temperature and *T*’ the desired one, expressed in kelvin (K):

A = (0.51 + (0.856·(*T*’ − 298.15) + 0.00385·(*T* − 298.15)^2^)/1000) (7)

and

L = (∆H_n_^0^ − z*·(1.5 + 0.024·(*T*’ − 298.15)·√*I*)/(1 + 1.5·√*I*)) (8)

By using Equation (8), it is possible to calculate, the standard enthalpy changes value of formation of the species at infinite dilution ∆H_n_^0^ and in the investigated Δ*T* range. Equation (8) takes also into account the variation of the ∆H_n_^0^ with *I*/mol dm^−3^.

#### 2.4.2. Specific Ion Interaction Theory (SIT) Approach

When the stability constants and the ionic strengths are expressed in the molal concentration scale, Equation (6) becomes (neglecting the last term containing ∆H_n_^0^) the classical and widely used Specific Ion Interaction Theory (SIT) equation [20,21,22], in which C is replaced by ∆ε, as follows:

logβ_pqr_ = log^T^β_pqr_ − z* (A√(*I*_m_))/(1 + 1.5·√*I*_m_) + ∆ε·*I*_m_ + jloga_w_ (9)

where *I*_m_ = ionic strength in the molal concentration scale, log a_w_ is the activity coefficient of water (log a_w_ = 0.015), j = number of water molecules involved in the equilibrium.

∆ε = ∑ε_reactants_ − ∑ε_products_ (10)

ε is the SIT coefficient for the interaction of the ionic species involved in the considered equilibrium with the ion (of opposite sign) of the supporting electrolyte (i.e., NaCl).

In all the equilibria involving uncharged species, the activity coefficient must also be taken into account by means of the Setschenow equation [31].

Log γ = k_c,m_ · *I* (11)

where k_c_ and k_m_ are the Setschenow coefficients of the neutral species in a given medium, in the molar and molal concentration scales, respectively.

If all interactions between the ligand and the metal ion are taken into account, the generic ∆ε parameter of Equation (9) can be explicated to obtain the ion-pairs SIT coefficient for all species involved in the equilibrium of formation of the complexes. As an example, in the case of the ML species, we have for M^2+^ (Ca^2+^, Mg^2+^) or M^+^ (CH_3_Hg^+^) cations:

M^2+^: ∆ε = ε(M^2+^,Cl^−^) + ε(L^−^,Na^+^) − ε(ML^+^,Cl^−^) (12)

M^+^: ∆ε = ε(M^+^,Cl^−^) + ε(L^−^,Na^+^) − k_m_(ML^0^) (13)

For the MLH species and M^2+^ cation, we have as an example:

∆ε = ε(M^2+^,Cl^−^) + ε(L^−^,Na^+^) + ε(H^+^,Cl^−^) − ε(MLH^2+^,Cl^−^) (14)

For MLOH species and M^2+^ cation, if the following equilibrium of formation is considered, M^2+^ + L^−^ + H_2_O = MLOH^0^ + H^+^

The specific ion interaction coefficients can be calculated by:

∆ε = ε(M^2+^,Cl^−^) + ε(L^−^,Na^+^) − k_m_(MLOH^0^) − ε(H^+^,Cl^−^) (15)

In this case, the activity coefficient of water must be considered (log a_w_ = 0.015).

## 3. Results

### 3.1. Acid-Base Properties of Dopamine and Hydrolytic Constants of the Investigated Cations

The hydrolytic constants of the cations here investigated, and the protonation constants of dopamine were already investigated.

In a previous paper [10], the protonation constants of dopamine in aqueous solutions of sodium chloride were determined at different ionic strengths and temperatures.

The hydrolysis constants of CH_3_Hg^+^ in NaCl at 0 < *I*/mol dm^−3^ ≤ 1.0 were taken from previous investigations [23,32]. In chloride media, the acid-base behavior of methylmercury(II) is characterized by the formation of a stable CH_3_HgCl complex that forms in significant amount and represents the main species up to pH~7–8.

For Mg^2+^, the hydrolytic constants were taken from [33]; whilst for Ca^2+^ from [34].

For Sn^2+^, the acid-base properties were already studied in a previous work, from an accurate analysis of literature data and experimental investigations, at different conditions (i.e., *I*/mol dm^−3^, *T*/K, etc.) in NaCl aqueous solutions [35], by also determining the stability of the weak complexes with chloride anion. The literature data for the protonation constants of dopamine, the hydrolysis of the metal ions and the corresponding stability constants of the complexes formed with Cl^−^ are reported in Tables S1 and S2 of the Supplementary Material.

### 3.2. CH_3_Hg^+^/Dop^−^ System

The determination of the best speciation model for the metal/dopamine systems and of the corresponding stability constants was obtained by using some general rules and guidelines, already applied in previous papers [18,24,36].

Investigations regarding the methylmercury/dopamine system proved to be particularly complex. These complexities can be explained by taking into consideration two factors: the first is related to the experimental measurements carried out in aqueous solutions of NaCl, the second one to the tendency of this metalloid to form a complex of high stability with the chloride, avoiding the hydrolysis of CH_3_Hg^+^, up to rather high pH values (~8).

The interactions of dopamine towards methylmercury(II) was investigated in quite wide experimental conditions, from *T* = 288.15 to 318.15 K and from *I* = 0.15 to 1.0 mol dm^−3^. In order to check the formation of all the possible cation/ligand complexes, solutions at different metal:ligand molar ratios were prepared (Table 2).

The potentiometric titrations were carried out up to pH~10, without observing the formation of sparingly soluble species. The stability constants of CH_3_Hg^+^/Dop^−^ species at different ionic strengths and temperatures are reported in Table 3.

**Figure 2:**
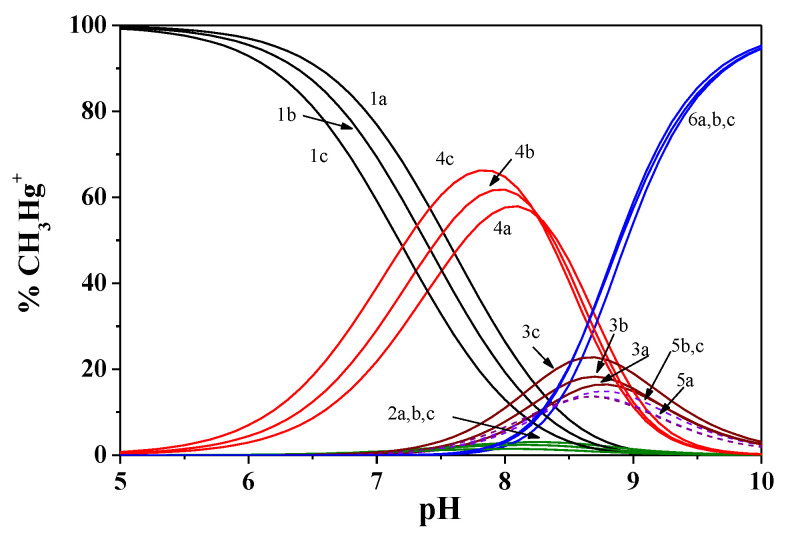
Distribution diagram for the CH_3_Hg^+^/Dop^−^ species at *T* = 298.15 K and different ionic strengths. Species: 1. MCl; 2. MOH; 3. ML; 4. MLH; 5 MLCl; 6. MLOH. a: *I* = 0.151 mol dm^−3^; b: *I* = 0.502 mol dm^−3^; c: *I* = 1.002 mol dm^−3^. (Experimental conditions: *c*_CH3Hg_^+^ = 0.5 mmol dm^−3^; *c*_DOP_^−^ = 1.5 mmol dm^−3^) [M = CH_3_Hg^+^; L = Dop^−^].

As already observed in previous investigations [18,23,32], the speciation of methylmercury(II) in sodium chloride aqueous solutions is characterized by the formation of the stable MCl species (Table S2 of the Supplementary Material), so that in the presence of dopamine, the formation of the ternary MLCl complex, together with the ML, MLH, and MLOH ones, is observed, as reported in the speciation diagram in Figure 2.

In particular, we observe an increase of the formation percentage of the MLCl species with increasing the chloride concentration (i.e., the ionic strength) in the solution, and a formation percentage that reaches ~20% at *I* = 1.0 mol dm^−3^.

The other CH_3_Hg^+^/Dop^−^ species form with a yield of 15–20% for the ML in dependence on the ionic strength, and of ~90–95% for the MLOH at pH~9.5–10.

At *I* = 0.15 mol dm^−3^, and between pH~6–9, the MLH is the main species of the CH_3_Hg^+^/Dop^−^ system, reaching a formation of 55–65% at pH~8. Increasing the ionic strength, the maximum of formation of MLH shifts at lower pH, reaching ~70% at *I* = 1.0 mol dm^−3^. The profile of the MLOH curves is instead almost unchanged. This behavior could seem strange, but it can be easily explained considering that the overall formation constant of this species is characterized by z* and p* values equal to zero (see Section 2.4.1).

**Figure 3:**
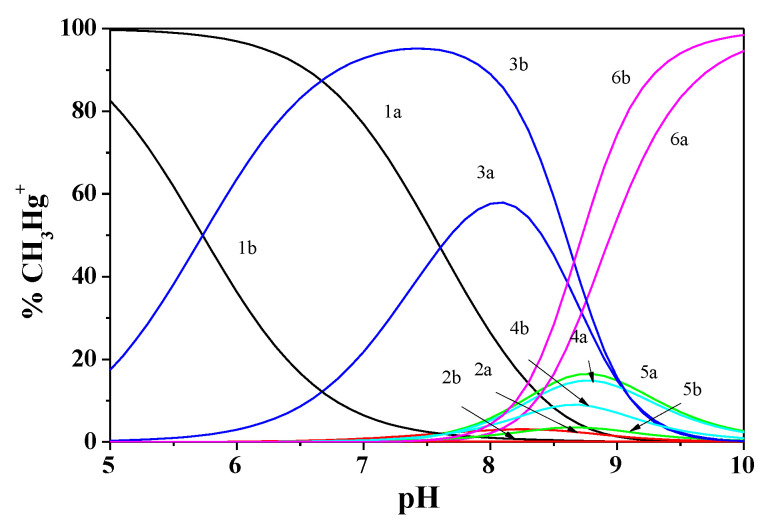
Distribution diagram for the CH_3_Hg^+^/Dop^−^ species at *I* = 0.151 mol dm^−3^ and different temperatures. Species: 1. MCl; 2. MOH; 3. MLH; 4. ML; 5 MLCl; 6. MLOH. a: *T* = 298.15 K; b: *T* = 318.15 K. (Experimental conditions: *c*_CH3Hg_^+^ = 0.5 mmol dm^−3^; *c*_DOP_^−^ = 1.5 mmol dm^−3^) [M = CH_3_Hg^+^; L = Dop^−^].

The effect of the temperature on the speciation and distribution of the CH_3_Hg^+^/Dop^−^ species is shown in the Figure 3.

Some important aspects can be discussed; the first one is that the MCl species undergoes a considerable shift on the maximum of formation, changing the temperature, as well as the corresponding formation percentage. As an example, at pH ~ 6.5, we have percentages of ~18% at *T* = 318.15 K and ~90% at *T* = 298.15 K. The opposite trend is observed for the ternary MLOH and MLH species. In the case of ML and MLCl, the pH ranges of formation remain almost unchanged, while the percentages of formation are lower increasing the temperature.

### 3.3. Metal^2+^/Dop^−^ Systems

The complexing ability of dopamine towards the bivalent cations (Mg^2+^, Ca^2+^, and Sn^2+^) was investigated at different component concentrations and metal:ligand molar ratios, as already reported in Table 2. Owing to the quite different acid-base properties of Ca^2+^ and Mg^2+^ with respect to Sn^2+^, different speciation models and stability of the complex species were obtained.

#### 3.3.1. Ca^2+^/Dop^−^ System

For the calcium interactions with dopamine, the speciation model, which gave the best results in terms of statistical parameters, was characterized by only the MLH and MLOH species. At *T* = 298.15 K, the higher number of available experimental data collected at different ionic strengths also allowed the determination of the ML species, which is formed in smaller percentages if compared with the other two MLH and MLOH complexes.

The formation constants for the Ca^2+^/Dop^−^ species are reported in Table 4; in particular, data at *T* = 298.15 K, refer to different ionic strengths, whilst at *T* = 288.15 and 310.15 K, only to *I* = 0.15 mol dm^−3^. From the results, we can observe a low stability for each species.

**Figure 4:**
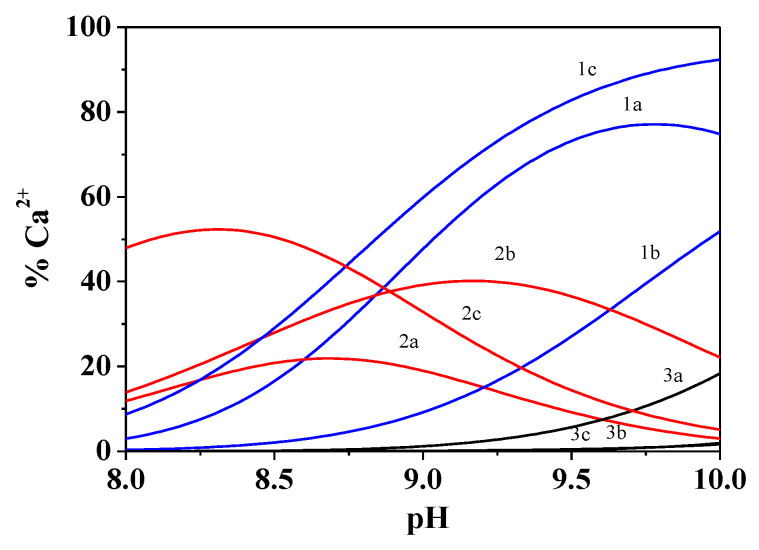
Distribution diagram for the Ca^2+^/Dop^−^ species at *T* = 298.15 K and different ionic strengths. Species: 1 ML; 2 MLH; 3 MLOH. a: *I* = 0.143 mol dm^−3^; b: *I* = 0.472 mol dm^−3^; c: *I* = 0.933 mol dm^−3^. (Experimental conditions: *c*_Ca_^2+^ = 1.5 mmol dm^−3^; *c*_DOP_^−^ = 5 mmol dm^−3^) [M = Ca^2+^; L = Dop^−^].

**Figure 5:**
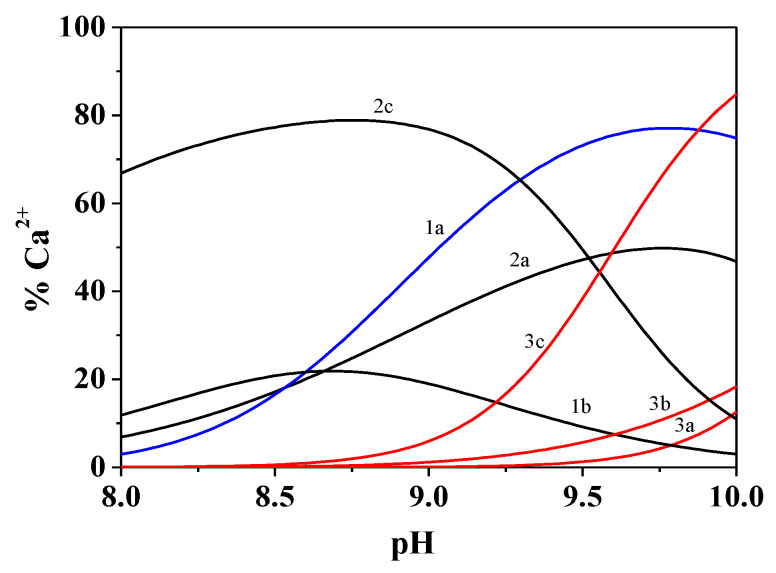
Distribution diagram for the Ca^2+^/Dop^−^ species at *I*~0.15 mol dm^−3^ and different temperatures. Species: 1 ML; 2 MLH; 3 MLOH.a: *T* = 288.15 K; b: *T* = 298.15 K; c: *T* = 310.15 K. (Experimental conditions: *c*_Ca_^2+^ = 1.5 mmol dm^−3^; *c*_DOP_^−^ = 5 mmol dm^−3^) [M = Ca^2+^; L = Dop^−^].

Some interesting observations can be made from the distribution diagrams given in Figure 4 and Figure 5. The complexation takes place at pH > 7.5, except for the ionic strength *I* = 1.0 mol dm^−3^, where it begins at lower pH.

As it can be highlighted in Figure 4, the useful range for the complexation is just two units of pH, since at pH~10, the formation of an insoluble species is observed.

The distribution of the Ca^2+^/Dop^−^ species at different temperatures is reported in Figure 5. We can observe a significant variation on the distribution of the species, both in terms of the pH of maximum formation and formation percentages.

#### 3.3.2. Mg^2+^/Dop^−^ System

The Mg^2+^/Dop^−^ interactions were investigated at different of ionic strengths (*I* range = 0.15–1.0 mol dm^−3^) and temperatures (from *T* = 288.15 to 310.15 K). The stoichiometry and stability constants of the Mg^2+^/Dop^−^ species were determined following the procedure already reported in the previous sections. The selected speciation model that gave the best results in terms of statistical parameters considers the ML and MLOH species.

Table 5 reports the formation constants at different temperatures and ionic strengths in NaCl aqueous solution.

The formation of the metal–ligand species occurs at pH~8.5, and up to this pH value magnesium is present as free metal ion, and only at higher pH, the MLOH(aq) complex becomes the predominant one, and ML reaches ~10%.

As an example, the distribution of the Mg^2+^/Dop^−^ species at *T* = 310.15 K and *I* = 0.15 mol dm^−3^ is reported in Figure S1 of the Supplementary Material.

Unlike the Ca^2+^/Dop^−^ system, the formation of the MLH species was not observed. This can be probably due to the different behavior of the two metals in term of hydrolytic constants.

As an example, from the distribution diagrams of the two metal/Dop systems, we observe that the Ca^2+^ and dopamine interaction begins at pH~7.5, whilst for Mg^2+^, at about 8.5; this aspect can explain why, in the second case, we did not obtain the formation of the MLH species.

#### 3.3.3. Sn^2+^/Dop^−^ System

Sn^2+^ has different acid-base properties and behaviors with respect to calcium and magnesium, since it tends to form polynuclear hydrolytic species also at low pH values [18,37,38,39]. Moreover, Sn^2+^ forms, SnCl_i_ (i = 1–3) species of significant stability and a mixed SnOHCl hydrolytic complex in chloride solutions. As already reported in a previous investigation [35], the formation of the Sn(OH)_2_(s) sparingly soluble species at pH values is dependent on the metal concentration, ionic medium and ionic strength and must be considered. All the equilibria of formation of these species, together with the dopamine protonation constants, were used as input in the speciation scheme. Applying the already cited criteria of selection of the speciation model, the best results were obtained when the following complexes were considered: M_2_L_2_, ML_2_, MLOH, and M_2_LOH, whose stability constants are reported in Table 6.

**Figure 6:**
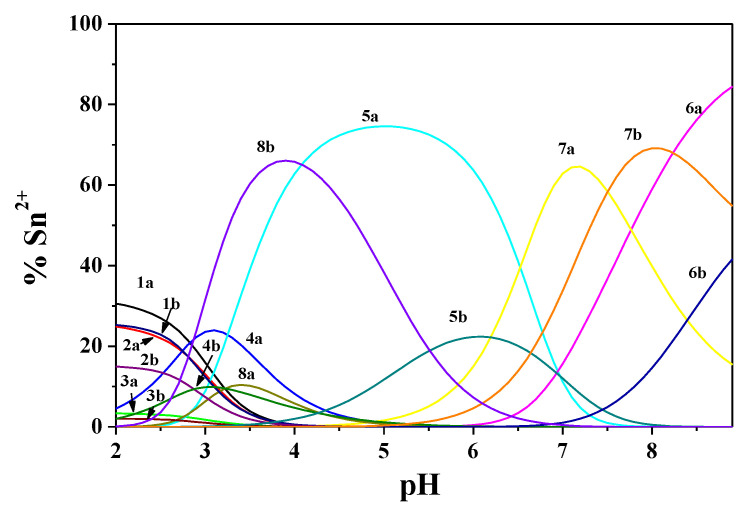
Distribution diagram for the Sn^2+^/Dop^−^ species a *T* = 298.15 K and different ionic strengths. a: *I* = 0.147 mol dm^−3^; b: *I* = 0.954 mol dm^−3^. Species: 1. MCl; 2. MCl_2_; 3. MCl_3_; 4. MCl(OH); 5. M_2_L_2_; 6. ML_2_; 7. MLOH; 8. M_2_LOH. (Experimental conditions: *c*_Sn_^2+^ = 1.5 mmol dm^−3^; *c*_DOP_^−^ = 5 mmol dm^−3^) [M = Sn^2+^; L = Dop^−^].

From the distribution diagrams shown in Figure 6, we can observe that all species are formed in high percentages, covering the entire range of pH investigated. Moreover, the free metal ion is present in very lower percentages (~5% and at pH < 4) with respect the other two M^2+^/Dop^−^ systems. The M_2_L_2_ species is present in almost all of the investigated pH ranges, representing the main complexes up to pH~6.5, beyond which the species MLOH and ML_2_ become the most important ones.

**Figure 7:**
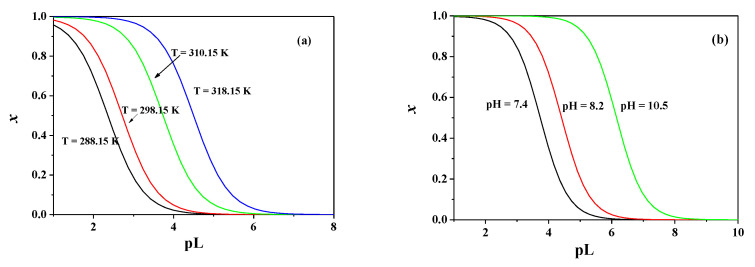
Sequestering diagram of dopamine towards CH_3_Hg^+^ in NaCl aqueous solution. (**a**) *I* = 0.15 mol dm^−3^, pH = 7.4 at different temperatures; (**b**) *I* = 0.15 mol dm^−3^, *T* = 310.15 K and different pH values. pL_0.5_ values: (**a**) 2.36 (*T* = 288.15 K) < 2.63 (*T* = 298.15 K) < 3.74 (*T* = 310.15 K) < 4.50 (*T* = 318.15 K). (**b**) 3.74 (pH = 7.4) < 4.41 (pH = 8.2) < 6.15 (pH = 10.5).

The strength of the Sn^2+^/Dop^−^ complexes avoids the formation of high amounts of the Sn_p_(OH)_q_ species, reaching about 5% of formation (not reported in Figure 7), even if the SnOHCl one at *I* = 0.15 mol dm^−3^ and pH~3, achieves about 20% of formation. The pH limit investigated (pH~8–9) is dependent on the different used experimental conditions and on the formation of an insoluble species. The effect of the ionic strength on the stability of the complexes can be highlighted observing the distribution of species at different pH values (Figure 6) and the corresponding formation percentages. Different formation percentages and a shift of the maximum of formation are observed when the ionic strength and the NaCl concentration increases from *I* = 0.15 to 1.0 mol dm^−3^. A similar observation can be made for the effect of the temperature; in this case, for the overall stability constants of M_2_L_2_ and ML_2_ species, differences of about 3 orders of magnitudes between *T* = 288.15 and 310.15 K at *I* = 0.15 mol dm^−3^ were observed. For the overall stability constants of MLOH and M_2_LOH, the differences are smaller, namely about 1 and 0.5 order of magnitudes, respectively.

### 3.4. Modeling of the Stability Constants with Respect to Ionic Strength and Temperature

As already reported in the Section 2.4, two different approaches can be used to model the dependence of the stability constants on the ionic strength.

The first approach uses an extended Debye–Hückel equation (Equation (6) of Section 2.4.1), which can also allow the modeling of the dependence of the stability constants on the temperature by the calculation of the enthalpy change values of formation of the metal/ligand complexes, when measurements at different *T*/K are performed. This equation was applied for the CH_3_Hg^+^/Dop^−^ and Mg^2+^/Dop^−^ systems, investigated at 288.15 ≤ *T*/K ≤ 318.15 and 0.15 ≤ *I*/mol dm^−3^ ≤ 1.0. For the modeling of the Ca^2+^ and Sn^2+^ systems, Equation (6) was employed, but without using the Van’t Hoff term, since experiments at different ionic strengths were only carried out at *T* = 298.15 K.

For these last two systems, the enthalpy change values of formation of the complex species were only calculated at *I* = 0.15 mol dm^−3^ by means of the classical formulation of the Van’t Hoff equation:

logβ_pqr_^T’^ = logβ_pqr_^T^ + 1/(R·2.303)·∆H^θ^·(1/*T* − 1/*T*’) (16)

where *T* and *T*’ are the reference and desired temperatures, respectively, expressed in kelvin (K); ∆H^θ^, is the temperature coefficient valid for the investigated temperature interval, that can be related to the standard enthalpy change values.

For CH_3_Hg^+^ and Mg^2+^ systems, the simplified Equation (6) (without considering the Van’t Hoff term) was also applied at each single temperature (Table 7 and Table 8).

By using the approaches above reported, the stability constants at infinite dilution and the parameters for the dependence on ionic strength were calculated, considering as reference temperature *T* = 298.15 K. These data are reported in Table 9 together with the enthalpy and entropy change values of formation of the species. We remember that the ∆H/kJ mol^−1^ and *T*∆S/kJ mol^−1^ of Ca^2+^ and Sn^2+^ systems refer to *I* = 0.15 mol dm^−3^.

From a comparison between the formation constants at the same experimental conditions, it is evident that Sn^2+^ forms complexes with dopamine featured by a higher stability, with respect to the other cations, and that it is the only metal that forms polynuclear complexes.

Concerning the second model used to study the dependence of the stability constants on the ionic strength, namely the SIT approach, both the concentrations and the stability constants were converted from the molar to the molal scale. The c/m ratio can be determined by the following formula:

c/m = d_0_ + a_1_c +a_2_c^2^ (17)

where d_0_ is the water density at *T* = 273.15 K, d_0_ = 0.99987 g/cm^3^. The dependence of water density on temperature can be modeled by the following equation:

d(*T*/K) = [(d_0_ + p_1_·*T*/K − p_2_·(*T*/K)^2^)/(p_3_·(*T*/K) + 1))]·10^−3^ (18)

where d(*T*/K) is the water density at the desired temperature; p_1_ = 8.119, p_2_ = 7.8735·10^−3^ and p_3_ = 8.0574·10^−3^ are empirical parameters obtained by modeling the data pair, pure water density (g/cm^3^) derived from literature data [40] vs. *T*/K. The knowledge of the water density at the desired temperature and the molarity and molality of the electrolytic solution (NaCl in our case) at a given saline concentration, allows the calculation of the c/m value. Using Equation (17), the following parameters have been calculated: a_1_ = −0.017765, a_2_ = −6.525·10^−4^. The range of validity for conversion in NaCl is: 0 ≤ *I* (NaCl)/mol kg^−1^ ≤ 6. The stability constants converted in the molal concentration scale, by using the above approach, are reported in Tables S3–S6 of the Supplementary Material.

From the ionic strength and formation constants expressed in the molal concentration scale, the application of the SIT model allows the determination of the specific ion interaction coefficients of all the ion pairs formed by the interactions of the cations with dopamine.

These calculations also require the use of the specific ion interaction coefficients of the other ionic species present in the solution, namely: ε(H^+^,Cl^−^) = 0.12 [41]; ε(Dop^−^,Na^+^) = −0.228 [10]; ε(Ca^2+^,Cl^−^) = 0.14 [29]; ε(Mg^2+^,Cl^−^) = 0.209 [29] and ε(Sn^2+^,Cl^−^) = 0.032 [35].

For the CH_3_Hg^+^/Dop^−^ system, this calculation is not possible, because the SIT coefficient for the interaction of methylmercury with chloride is unknown. In this case, the determination of Δε instead of classical SIT coefficients is particularly indicated, since the system of equations related to various equilibria results undetermined, hampering the calculation of other single ε. However, this approach was also applied to the other metal/dopamine systems here investigated.

The specific ion interaction coefficients (ε and ∆ε) of the ionic species and the Setschenow coefficients for the neutral ones are reported in Table 10.

### 3.5. Sequestering Ability of Dopamine towards Cations and Effect of pH, Ionic Strength, and Temperature

In studies concerning problems involving natural or biological fluids, it is often essential to have a parameter that allows estimating the ability of one or more ligands to sequester a given component, such as a metal ion. This estimation is particularly difficult when comparing two different systems or the same system studied at different temperatures, ionic strengths, and ionic media. When the systems to be compared also have different speciation and only a few (or none) are the common species among them, understanding which systems have higher or lower sequestering ability is fairly impossible. This problem is even more complex if the system of interest is contained in a multicomponent solution, where the possible simultaneous presence of several metal and/or organic and inorganic components makes this study more complex. For example, if we want to calculate the sequestering ability of a given M^n+^ metal with an organic ligand L^z−^, the metal may undergo hydrolysis reactions and form complexes with the anions of the ionic medium or with other competing ligands.

The ligand L^z−^ in turn can be present in its different protonate or deprotonate forms and reacts with the cation of the ionic medium or with other metals. Therefore, it is essential to use a parameter that can allow the calculation of the sequestering ability of a ligand towards a metal ion at different experimental conditions.

Many years ago, this research group introduced the pL_0.5_ parameter, which, once the conditions of pH, ionic strength, ion medium, and temperature are established, gives an objective representation of the sequestering ability towards one or more metals [23,24]. It is determined by applying a Boltzmann type equation to the couple of data: the sum of the mole fraction of all the formed metal–ligand species vs. the logarithm-changed sign of the ligand analytical concentration (pL).The proposed equation is:

x = 1/(1 + 10^(pL−pL0.5)^) (19)

where x is the total fraction of complexed metal plotted versus pL, (pL = −log [L]_tot_ and [L]_tot_ is the analytical concentration of ligand). This function is like a sigmoidal curve with an asymptote of 1 for pL→−∞ and 0 for pL→+∞. pL_0.5_ is a quantitative parameter and represents the concentration of ligand required for the sequestration of 50% of the metal cation. It is important to remember that this property varies as experimental conditions change, but is independent of the analytical concentration of metal when it is present in traces (~10^−10^ mol dm^−3^ or less). In the calculation of pL_0.5_, all the parallel reactions (metal hydrolysis, ligand protonation, reactions with other components, etc.) are considered in the speciation model, but are successively excluded in the calculation of pL_0.5_.

Using this approach, the dopamine sequestering ability against the metal cations here investigated was determined at different experimental conditions (*I*/mol dm^−3^, pH, *T*/K), and the pL_0.5_ values are reported in Table 11, Table 12 and Table 13.

**Figure 8:**
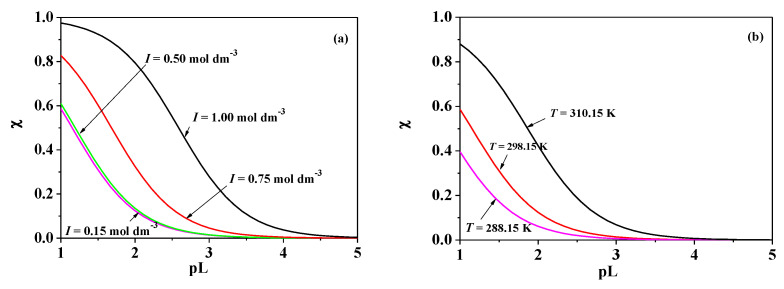
Sequestering diagram of dopamine towards Ca^2+^. (**a**) *T* = 298.15 K, pH = 7.4 and different ionic strengths; (**b**) *I* = 0.15 mol dm^−3^, pH = 7.4 and different temperatures. pL_0.5_ values: (**a**) 1.15 (*I* = 0.15 mol dm^−3^) < 1.19 (*I* = 0.50 mol dm^−3^) < 1.68 (*I* = 0.75 mol dm^−3^) < 2.58 (*I*= 1.0 mol dm^−3^). (**b**) 0.81 (*T* = 288.15 K) < 1.15 (*T* = 298.15 K) < 1.86 (*T* = 318.15 K).

**Figure 9:**
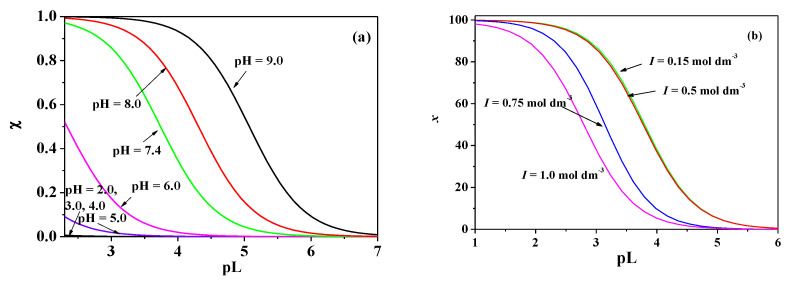
Sequestering diagram of dopamine towards Sn^2+^. (**a**) *T* = 298.15 K, *I* = 0.15 mol dm^−3^ and different pHs. (**b**) *T* = 298.15 K, pH = 7.4 and different ionic strengths. pL_0.5_ values: (**a**) 1.30 (pH = 5.0) < 2.33 (pH = 6.0) < 3.74 (pH = 7.4) < 4.31 (pH = 8.0) < 5.07 (pH = 9.0). (**b**) 3.74 (*I* = 0.15 mol dm^−3^) < 3.70 (*I* = 0.50 mol dm^−3^) < 3.12 (*I* = 0.75 mol dm^−3^) < 2.60 (*I* = 1.0 mol dm^−3^).

**Figure 10:**
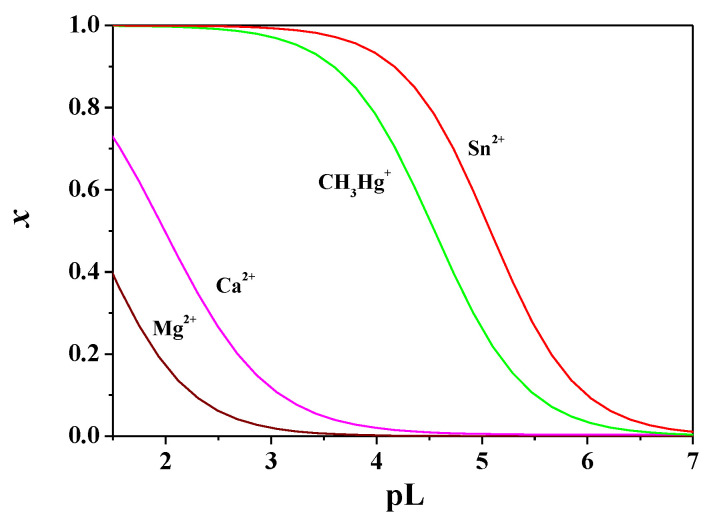
Sequestering diagram of dopamine towards the metal ions here investigated, at *I* = 0.15 mol L^−1^, *T* = 298.15 K, and pH = 9.0.

**Figure 11:**
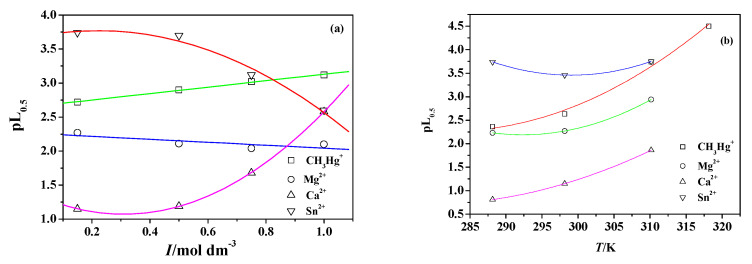
Dependence of pL_0.5_ on: (**a**) *I*/mol dm^−3^ and (**b**) *T*/K. (**a**) pL_0.5_ values calculated at *T* = 298.15 K, pH = 7.4 and different ionic strengths; (**b**) pL_0.5_ values calculated at *I* = 0.15 mol dm^−3^, pH = 7.4 and different temperatures.

For a better visualization of the results, some sequestering diagrams have been drawn (Figure 8, Figure 9, Figure 10 and Figure 11).

From the sequestering diagrams here reported, it is possible to observe that the sequestering ability of dopamine towards the metals is dependent both on the ionic strength values, on the temperature, and pH. The increase of the pL_0.5_ with pH (Figure 7) is related to the deprotonation of the ligand and an indication of the electrostatic nature of the interactions. At the same pH, the dependence of dopamine sequestering ability on the ionic strength (Figure 8) is due both to the metal acid-base property, the strength of interaction between the components, the tendency of the metal to form hydrolytic species and stable weak complexes with the anion of the ionic medium, which contribute to reduce the amount of free metal ion to interact with the ligand.

In Figure 9, the effect of pH and saline concentration on the pL_0.5_ values of the Sn^2+^/Dop^−^ system is reported. Concerning the ionic strength influence, (Figure 9b) ∆pL_0.5_ values of ~1.14 units from *I* = 0.15 to 1.0 mol dm^−3^ was obtained. With respect to Ca^2+^/Dop^−^ system, we can observe an opposite trend, namely the increase of the ionic strength (i.e., NaCl concentration) produces a lowering of pL_0.5_. This effect can be explained considering that increasing the Cl^−^ concentration, the formation of the SnCl_i_ (i = 1–3) and SnOHCl species is favorite, contributing to a lowering of the Sn^2+^ free concentration.

Similarly, temperature gradients also affect the ligand sequestering ability towards metals. Table 13 compares the pL_0.5_ values of the different systems, at different temperatures.

In this case, independent of the system and pH range investigated, an increasing trend of the pL_0.5_ values with increasing the temperature is observed.

In order to evaluate the sequestering ability of dopamine towards the metal ions under study, the pL_0.5_ values were calculated at the same experimental conditions, i.e., *T* = 298.15 K, *I* = 0.15 mol dm^−3^ and pH = 9.0 (but similar results can be obtained from a comparison between all the data reported in Table 13, Table 14 and Table 15). From the pL_0.5_ values reported in Figure 10, the following sequestering ability trend of dopamine towards the investigated metals can be observed:

pL_0.5_: Mg^2+^ < Ca^2+^ < CH_3_Hg^+^ < Sn^2+^

**Figure 12:**
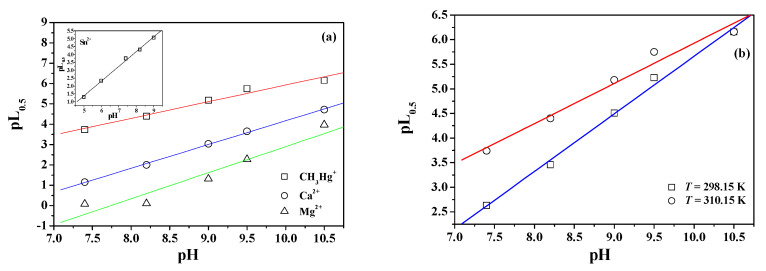
Dependence of pL_0.5_ on pH. (**a**) pL_0.5_ values calculated at *T* = 298.15 K and *I* = 0.15 mol dm-3; (**b**) pL_0.5_ values of CH_3_Hg^+^/Dop^−^ system calculated at *I* = 0.15 mol dm^−3^, pH = 7.4, and *T* = 298.15 and 310.15 K.

The effect of the variables, *T*/K, *I*/mol dm^−3^ and pH on the sequestering ability of dopamine towards the metal ions can be also observed in Figure 11 and Figure 12.

pL_0.5_: Mg^2+^ = 1.31 < Ca^2+^ = 2.00 < CH_3_Hg^+^ = 4.58 < Sn^2+^ = 5.08.

Figure 11a, shows the variation of the pL_0.5_ values with respect to ionic strength; for Mg^2+^ and CH_3_Hg^+^, a linear trend, even if opposite, is observed increasing the ionic strength. For Ca^2+^ and Sn^2+^, a non-linear variation is reported, and also in this case with an opposite trend.

The net increase of pL_0.5_ with the ionic strength is an indication of a stabilizing effect of the ion coming from the ionic media on the stability of the complexes. In particular, for CH_3_Hg^+^, the trend can be explained observing the distribution diagram reported in Figure 2, where increasing the ionic strength, there is an increase of the formation percentage of the MLCl species.

The opposite effect was observed for Sn^2+^ (see also Figure 6). In this case, the decrease of the pL_0.5_ values can be explained considering that increasing the ionic strength, a lowering of the formation percentage of the species can be observed, especially at pH > 7, where the percentage of formation of the ML_2_ decreases from ~22% at *I* = 0.15 mol dm^−3^ to ~5% at *I* = 1.0 mol dm^−3^. Figure 11b reports the effect of the temperature on pL_0.5_, obtaining an increasing trend. In Figure 12a,b, the effect of the variation of the pH on the sequestering ability of dopamine towards the metals is highlighted. For CH_3_Hg^+^ (Figure 12b), the effect of the temperature is less evident, and increasing the pH up to ~10, the differences are fairly nil.

Considering the amount of available data, the dependence of pL_0.5_ on the variables (*I*/mol dm^−3^, *T*/K, and pH) was modeled by means of the LIANA computer program. For each metal/dopamine system, the best results in term of statistical parameters, were obtained when the following simple empirical equation was used:

pL_0.5_ = p_1_ + p_2_·*T*/K + p_3_·(*I*/mol dm^−3^)^2^ + p_4_·pH (20)

where p_i_ = 1–4 are the empirical parameters.

The values of the p_i_ parameters and the corresponding standard and mean deviation on the fit are reported in Table 14.

### 3.6. Thermogravimetric Analysis

Some thermogravimetric analyses were performed, by using a PerkinElmer thermogravimetric balance model Pyris Diamond (PerkinElmer, Milano, Italy), in oxidant purging atmosphere (air flow), in the temperature range *T* = 293.15–1073.15 K. These investigations were carried out on solid samples obtained at the end of potentiometry titrations of the Ca^2+^/Dop^−^ system and from solutions prepared at different metal to ligand molar ratios.

Before the TGA analysis, the samples, once collected, were filtered on 0.45 µm cellulose filters, and washed with small amounts of ultrapure water. Once washed, the precipitates were treated with small amounts of acetone and vacuum dried.

**Figure 13:**
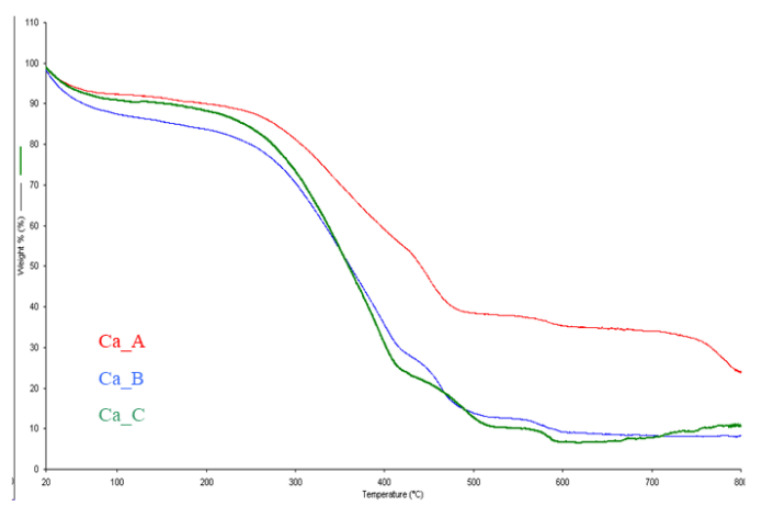
Thermogravimetric curve for the Ca^2+^/Dop^−^ precipitates obtained at different metal:ligand molar ratios and *I* = 0.15 mol dm^−3^. Component concentration in the solution: c_Ca_^2+^ = 1.5 mmol dm^−3^ and c_Dop−_ = 1.5–7.5 mmol dm^−3^. Atmosphere of air (gaseous mixture of nitrogen and oxygen with 80% and 20%, *v*/*v*, respectively) at a flow rate of 100 mL min^−1^ and a scanning rate of 10 K min^−1^.

**Figure 14:**
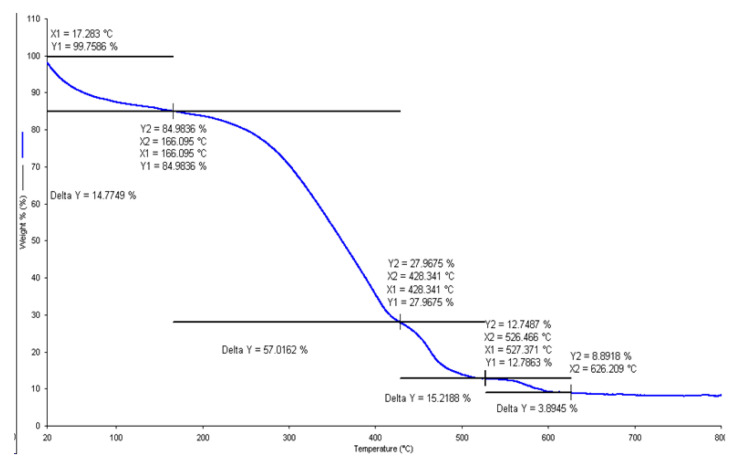
Thermogravimetric profile of the percentage weight loss for Ca^2+^/Dop^−^ B.

Figure 13 and Figure 14 report the thermogravimetric diagrams for three different Ca^2+^/Dop^−^ precipitates; it is possible to observe that the curves have different decomposition profiles with residual values at the end of analysis, where the metal oxide is present, clearly different.

Table 15 reports the percentage weight loss for each Ca^2+^/Dop^−^ system, calculated from the thermal decomposition of the precipitates. Considering these experimental data, the stoichiometry of the precipitates was calculated, assuming that the residual of the decomposition process is CaO. As it can be observed, the stoichiometry of the precipitate change between a metal:ligand molar ratios of 1:3 to 1:5, in dependence on the initial concentration of the components in the vessel.

## 4. Literature Data

Literature reports different papers where the interaction of dopamine towards metal ions has been investigated. However, to our knowledge, no thermodynamic studies on the interaction with the cations under investigation have ever been reported, except for Mg^2+^. The stability constants for the systems published in the literature are listed in Table 16. The main difficulty for a direct comparison of these data with the results here obtained is the different approaches used for the determination of the acid-base properties of dopamine. In some cases, the ligand was considered as a triprotic ligand (L^2−^), by the calculation of the third protonation constant having a log *K*^H^ value of about 13–14, out of the pH range of physiological interest. In other cases, only the protonation constants of one phenolic group and of the amine group of the alkyl chain were considered.

The literature stability constants reported in Table 16, refer to low ionic strength values (*I* ≤ 0.2 mol dm^−3^), and this aspect does not allow a comparison with the stability constants here reported up to *I* = 1.0 mol dm^−3^, as well as for the dependence on the ionic strength and temperature.

The only attempt of comparison that can be done between our and literature data regards the speciation models. They are mainly formed by simple mononuclear species, such as: ML, ML_2_, and some ternary protonated ones. For the systems reported in the literature, the formation of metal polynuclear species, such as M_2_L_2_ and M_2_LOH, obtained for Sn^2+^, is not observed.

A fairly good agreement between our and the literature data there is for Mg^2+^, logβ_ML_ = 4.57–4.49 at *I* = 0.2 mol dm^−3^ in NaCl aqueous solution and different temperatures (from *T* = 288.15 to 308.15 K) [42].

As an example, from our investigation we obtained at *I* = 0.15 mol dm^−3^ and *T* = 298.15 K, logβ_ML_ = 3.034 ± 0.044. The difference with respect the literature value can be explained considering that our speciation model also includes the MLOH species, which becomes significant at pH values higher than 8.5, and cannot be neglected in the speciation of the system. The formation constant of the ternary MLOH hydrolytic species is: logβ_MLOH_ = −6.111 ± 0.016 at *I* = 0.15 mol dm^−3^ and *T* = 298.15 K.

Another attempt of comparison can be made for the CH_3_Hg^+^/Dop^−^ complexes here determined and those already reported in a previous investigation for the CH_3_Hg^+^/Eph^−^ (adrenaline) interactions [18]. A similar speciation model has been obtained; if we compare, as a pure example, the stability of the complexes at *I* = 0.15 mol dm^−3^ and *T* = 298.15 K in NaCl aqueous solution, for adrenaline we have the following values: logβ_ML_ = 8.56 (10.43 for Dop); logβ_MLH_ = 17.33 (19.43 for Dop); logβ_MLOH_ = −0.79 (1.99 for Dop) and logβ_MLCl_ = 9.17 (11.21 for Dop). It is possible to observe that dopamine forms more stable complexes with CH_3_Hg^+^ with respect to adrenaline, with a difference of about of two orders of magnitude.

However, the difficulty to carry out an accurate comparison between our and literature systems, in terms of stability of the species, is also due to the apparent neglection from the different authors of the hydrolysis of the metals investigated. In fact, in those papers, authors report and discuss the acid-base properties of dopamine, but no information on the hydrolysis of the metal and their hydrolytic constants is reported. From our investigations, we have evidenced that the metal hydrolytic species coexist in some cases, especially in excess of metals, with the metal/dopamine complexes, as observed for Sn^2+^, where the Sn(OH)^+^ and Sn_3_(OH)_4_^2+^ species reach about 5% of formation in pH range values, where the formation of the Sn^2+^/Dop^−^ complexes occur, whilst the SnOHCl—about 20%.

**Figure 15:**
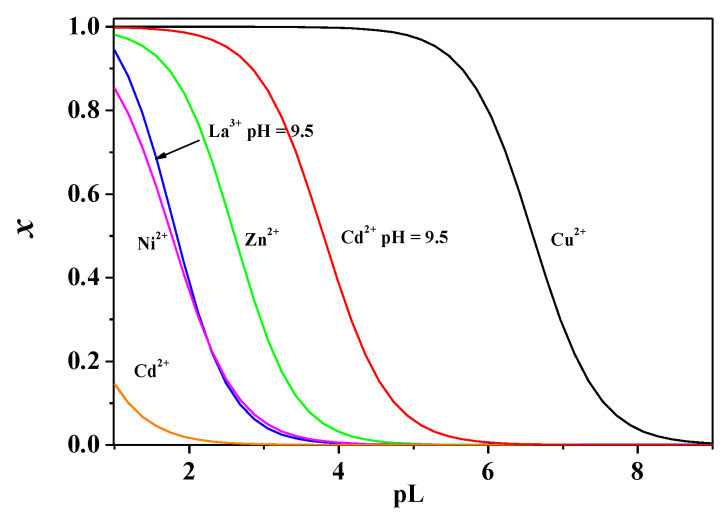
Sequestering diagram for some metal/dopamine systems, at *I* = 0.15 mol dm^−3^, *T* = 298.15 K and pH = 7.4.pL_0.5_: Cd^2+^ = 0.23; Ni^2+^ = 1.77; Zn^2+^ = 2.61; Cu^2+^ = 6.60 (pH = 9.5: La^3+^ = 1.85; Cd^2+^ = 3.80).

The use of the pL_0.5_ values can be useful to estimate the different sequestering ability of dopamine towards the metal ions. In this light, we selected the systems listed in Table 16, which reports the stability of the complexes determined at similar experimental conditions. By using the protonation constants proposed in those literature papers [44,45,48,49,50,51,52,53] and adding to the speciation models, the hydrolytic constants of the metals, taken from Brown and Edberg [54], the pL_0.5_ values were calculated at *T* = 298.15K, *I* = 0.2 mol dm^−3^ and pH = 7.4. The obtained values reported in Figure 15, can be compared with the values here presented for our systems.

From Figure 15, the following pL_0.5_ values have been obtained:

pL_0.5_: Cd^2+^ = 0.23; Ni^2+^ = 1.77; Zn^2+^ = 2.61; Cu^2+^ = 6.60.

For La^3+^, a negligible amount of metal was sequestered by dopamine; for this reason, the pL_0.5_ value was calculated at pH = 9.5, resulting to be pL_0.5_ = 2.85. As a pure comparison on the effect of the pH on the sequestration of Cd^2+^ by dopamine, also in this case the pL_0.5_ was calculated at pH = 7.4 and 9.5; the value obtained for Cd^2+^ are pL_0.5_ = 0.23 and 3.80, respectively. The very low pL_0.5_ value calculated for Cd^2+^ with respect to Zn^2+^ or Cu^2+^, can be explained considering that in chloride media, it forms quite stable CdCl_i_ (i = 1–3) complexes and the CdOHCl one, that reduce the free metal concentration for the interaction with the dopamine.

These literature data allow a merge with those here experimentally obtained at *I* = 0.15 mol dm^−3^ in NaCl aqueous solutions, *T* = 298.15 K and pH = 7.4; the following sequestering trend of dopamine towards the metals was obtained:

Mg^2+^ (0.08) < Cd^2+^ (0.23) < Ca^2+^ (1.15) < Ni^2+^ (1.77) < Zn^2+^ (2.61) < CH_3_Hg^+^ (2.63) < Sn^2+^ (3.74) < Cu^2+^ (6.60).

**Figure 16:**
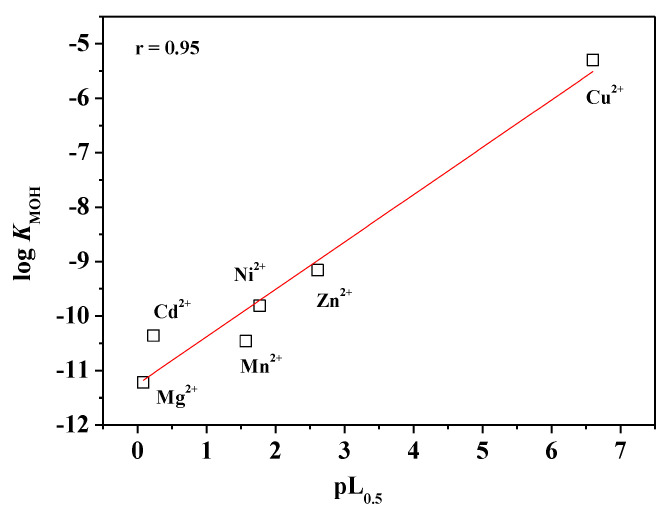
Relationship between the value log K_MOH_ value of some M^2+^ metal ions vs. pL_0.5_ calculated at *I* = 0.15 mol dm^−3^, *T* = 298.15 K and pH = 7.4.

Some simple correlations can be obtained from our and literature data; considering the pL_0.5_ values here calculated at *T* = 298.15 K and *I*~0.15–0.20 mol dm^−3^ (pH = 7.4), a simple fairly linear relationship is obtained with the first hydrolytic constant (M(OH) species) of the M^2+^ metal ions reported in Figure 16. The linear relationship is reported in Figure 16, where we observe a correlation coefficient r = 0.95.

**Figure 17:**
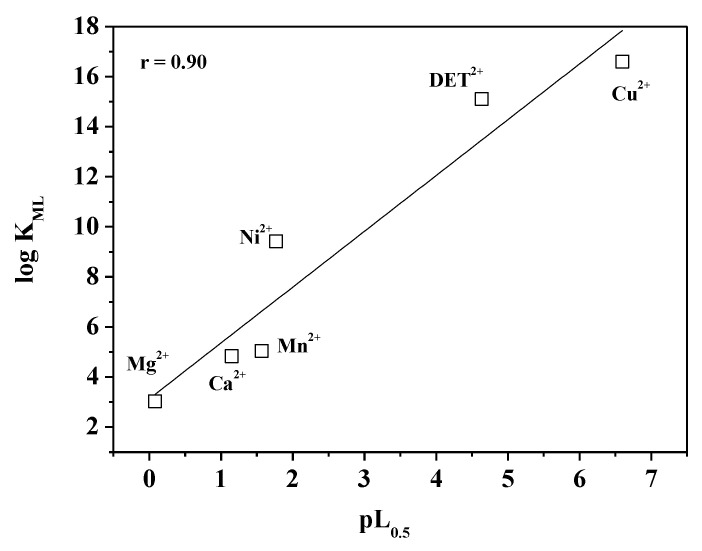
Relationship between the value log K_ML_ value of some M^2+^ metal/dopamine system vs. pL_0.5_. Data refer to *I* = 0.15 mol dm^−3^, *T* = 298.15 K and pH = 7.4 (for pL_0.5_ values).

Similar results can be obtained from the comparison of the pL_0.5_ values vs. the formation constants of the ML species of the M^2+^ metal/dopamine species, determined at *I* = 0.15 mol dm^−3^ and *T* = 298.15 K. Data for Mn^2+^ and (CH_3_CH_2_)_2_Sn^2+^ (DET) reported in Figure 17 are unpublished data from this research group. Moreover, for Figure 17, a fairly linear relationship (r = 0.90) was obtained.

Obviously, these correlations should be considered only as simple approximations since the pL_0.5_ also depends on many other variables, such as the other hydrolytic constants of the metal and the conditions where the pL_0.5_ is calculated, for example, pH, temperature, ionic strength, etc.

## 5. Discussion

The speciation studies carried out on dopamine interactions towards monovalent (CH_3_Hg^+^) and bivalent (Mg^2+^, Ca^2+^, and Sn^2+^) metal cations lead to the following conclusions:

1. Interactions with Ca^2+^ allow the formation of a simple speciation model with species of low stability.
2. Similar results were obtained for the Mg^2+^/Dop^−^ system, even if, in this case, the speciation model contains only the ML and MLOH species. The formation of the MLH species was checked at each experimental condition, but was always rejected by the computer program. The different speciation model with respect to Ca^2+^ can be attributed to the different acid-base properties of Mg^2+^ with respect to calcium. In fact, a comparison between the distribution diagrams of these two systems, highlight for Mg^2+^ a “complexation” with dopamine at higher pH values (pH~8.5) with respect to Ca^2+^ (pH~7.5), avoiding the formation of MLH.
3. The behavior of CH_3_Hg^+^ in NaCl aqueous solutions is also particular; in this case, the CH_3_HgCl species influences the speciation of the CH_3_Hg^+^/Dop^−^ system. In fact, the significant stability of the CH_3_HgCl species and the weakness of the complexes with dopamine lead to the formation of a mixed ternary complex (MLCl), which forms in a wide pH interval and at each investigated experimental condition.
4. Only for the Sn^2+^/Dop^−^ system, the processing of the experimental data highlights the formation of both mononuclear and binuclear complexes of medium-high stability, with variation of logβ_pqr_ values dependent on the ionic strength and temperature investigated.

The strength of the polynuclear complexes, also at low concentrations, avoid the formation of the simple ML ones, but allows the formation of the binary Sn_2_L_2_ species. This complex, together with the M_2_LOH ones, reaches high formation percentage along a wide pH interval.

5. The effect of the supporting electrolyte (NaCl) on the speciation of the metal/ligand systems can be observed for CH_3_Hg^+^ and Sn^2+^, since they tend to form stable complexes with chloride, influencing both the stability and distribution of the M^n+^/dopamine species and preventing the hydrolysis of the metal ions.
6. Measurements carried out at different ionic strengths and temperatures allowed to observe that the stability of the complexes is influenced by variation of these two variables, in particular that the entropy is generally the driving force of the formation of the metal/dopamine species.
7. The dependence of the stability constants on the ionic strength and temperature was modeled by means of different approaches (see Section 3.4), allowing the calculation of the Debye–Hückel parameters for the dependence on *I*/mol dm^−3^ and the specific ion interaction parameters (SIT) of the ion-pairs (and of the Setschenow coefficient for the neutral species) formed by the interaction of the metals with dopamine.
8. By using the pL_0.5_ parameter, it was possible to quantify, at different pH values, ionic strengths, and temperatures, the effective sequestering ability of dopamine towards the metals. This approach is very important, since the simple comparison of the stability constants values can lead to incorrect considerations, especially when comparing systems with different speciation and in different experimental conditions of temperatures, ionic strengths, pH values, or in different ionic media. The results here obtained highlight that the sequestering ability tends to vary as the temperature and ionic strength change. The sequestering ability of dopamine towards the metals follows the trend: pL_0.5_: Sn^2+^ > CH_3_Hg^+^ > Ca^2+^> Mg^2+^.
9. The precipitates collected at the end of potentiometric titrations were analyzed by thermogravimetric analysis (TGA) under a constant airflow (10 K/min). The analysis of the thermogravimetric profile of the curves allowed observing different stoichiometry of the solids, dependent on the different metal:ligand ratios, and concentrations of the solutions containing these components.
10. An analysis of the literature data allowed observing different speciation models and stability of the metal/dopamine complexes. The different sequestering ability of dopamine towards the metal ions was quantified by using the pL_0.5_ parameter, observing fairly linear relationships between this parameter and the first hydrolytic constants of the metals (log K_MOH_) or with the log K_ML_.

## Figures and Tables

**Table 1:** Chemicals used in this work, purchased from Sigma-Aldrich, Italy.

Chemical.	CAS Number	Purification Method	Purity (% wt.)	Purity Check
Hydrochloric acid	7647-01-0	none	≥99%	Volumetric titrations with Na_2_CO_3_
Potassium phthalate monobasic	877-24-7	none	≥99.5%	-
Sodium carbonate	497-19-8	none	≥99.5%	-
Sodium chloride	7647-14-5	none	≥99%	-
Sodium hydroxide	1310-73-2	none	≥99%	Volumetric titrations with potassium phthalate monobasic
CH_3_HgCl	115-09-3	none	≥99%	-
MgCl_2_·6H_2_O	7791-18-6	none	≥99%	Volumetric titrations with EDTA
CaCl_2_·2H_2_O	10035-04-8	none	≥99%	Volumetric titrations with EDTA
SnCl_2_·2H_2_O	10025-69-1	none	≥99%	Volumetric titrations with EDTA
Dopamine·HCl	62-31-7	none	≥99.5%	Volumetric titrations

**Table 2:** Experimental conditions used for the M^n+^/Dop^−^ systems in NaCl aqueous solutions.

c_M_ (mmol dm^−3^)	c_L_ (mmol dm^−3^)	M:L
1	1.5	1:1.5
1	4	1:4
1.5	3	1:2
1.5	5	1:3
2	5	1:2.5
2	6	1:3

M = generic metal ion; L = dopamine.

**Table 3:** Experimental formation constants of the CH_3_Hg^+^/Dop^−^ species in NaCl aqueous solutions at different ionic strengths and temperatures.

*I*/mol dm^−3^	logβ_ML_ ^a)^	logβ_MLH_ ^a^	logβ_MLCl_ ^a^	logβ_MLOH_ ^a^
		*T* = 288.15 K		
0.151	10.89 ± 0.05 ^b^	19.48 ± 0.08	11.42 ± 0.04	1.99 ± 0.06
0.502	10.87 ± 0.05	19.36 ± 0.09	11.32 ± 0.04	1.97 ± 0.06
0.751	10.91 ± 0.06	19.33 ± 0.11	11.31 ± 0.05	1.96 ± 0.06
1.004	10.95 ± 0.07	19.31 ± 0.13	11.30 ± 0.06	1.95 ± 0.07
		*T* = 298.15 K		
0.151	10.43 ± 0.03	19.43 ± 0.05	11.21 ± 0.02	1.99 ± 0.04
0.502	10.40 ± 0.03	19.30 ± 0.07	11.10 ± 0.03	1.98 ± 0.04
0.748	10.44 ± 0.04	19.27 ± 0.09	11.09 ± 0.04	1.97 ± 0.05
1.002	10.48 ± 0.06	19.25 ± 0.11	11.08 ± 0.05	1.96 ± 0.06
		*T* = 310.15 K		
0.152	9.91 ± 0.05	19.37 ± 0.03	10.96 ± 0.01	2.01 ± 0.02
0.496	9.88 ± 0.05	19.24 ± 0.06	10.86 ± 0.03	1.99 ± 0.03
0.751	9.91 ± 0.06	19.20 ± 0.09	10.84 ± 0.04	1.98 ± 0.03
0.998	9.95 ± 0.07	19.18 ± 0.12	10.83 ± 0.05	1.97 ± 0.05
		*T* = 318.15 K		
0.150	9.58 ± 0.07	19.33 ± 0.04	10.81 ± 0.02	2.01 ± 0.02
0.495	9.54 ± 0.07	19.19 ± 0.06	10.70 ± 0.03	1.99 ± 0.02
0.751	9.57 ± 0.07	19.16 ± 0.09	10.68 ± 0.04	1.99 ± 0.03
1.002	9.61 ± 0.08	19.13 ± 0.12	10.67 ± 0.06	1.98 ± 0.05

^a^ Refers to the general equilibrium: pM^n+^ + qL^z−^ + rH^+^ + Cl^−^ = M_p_L_q_H_r_Cl^(np−zq+r−1)^; ^b^ ±Std. Dev.

**Table 4:** Experimental formation constants of the Ca^2+^/Dop^−^ species in NaCl aqueous solutions.

*T*/K	*I*/mol dm^−3^	logβ_ML_ ^a^	logβ_MLH_ ^a^	logβ_MLOH_ ^a^
288.15	0.150	-	13.60 ± 0.03 ^b^	−6.97 ± 0.08 ^b^
298.15	0.143	4.83 ± 0.08 ^b^	13.43 ± 0.08	−5.78 ± 0.16
298.15	0.472	3.65 ± 0.07	13.28 ± 0.06	−7.78 ± 0.10
298.15	0.692	3.95 ± 0.07	13.65 ± 0.05	−6.69 ± 0.09
298.15	0.933	5.26 ± 0.10	14.44 ± 0.06	−6.49 ± 0.15
310.15	0.150	-	13.51 ± 0.23	−5.60 ± 0.22

^a^ Refers to the general equilibrium: pM^n+^ + qL^z−^ + rH^+^ = M_p_L_q_H_r_^(np−zq+r)^; ^b^ ±Std. Dev.

**Table 5:** Experimental formation constants of the Mg^2+^/Dop^−^ species in NaCl aqueous solutions at different ionic strengths and temperatures.

*I*/mol dm^−3^.	logβ_ML_ ^a)^	logβ_MLOH_ ^a)^
	*T* = 288.15 K	
0.151	3.459 ± 0.068 ^b)^	−6.278 ± 0.040 ^b)^
0.503	3.259 ± 0.078	−6.676 ± 0.030
0.753	3.267 ± 0.084	−6.905 ± 0.032
1.004	3.467 ± 0.092	−7.336 ± 0.012
	*T* = 298.15 K	
0.148	3.034 ± 0.044	−6.111 ± 0.026
0.501	2.797 ± 0.052	−6.531 ± 0.020
0.753	2.777 ± 0.056	−6.825 ± 0.022
0.996	2.951 ± 0.092	−7.324 ± 0.008
	*T* = 310.15 K	
0.150	2.561 ± 0.064	−5.926 ± 0.040
0.496	2.281 ± 0.074	−6.369 ± 0.030
0.748	2.232 ± 0.082	−6.736 ± 0.034
1.003	2.375 ± 0.092	−7.311 ± 0.012

^a)^ Refers to the general equilibrium: pM^n+^ + qL^z−^ + rH^+^ = M_p_L_q_H_r_^(np−zq+r)^; ^b)^ ±Std. Dev.

**Table 6:** Experimental formation constants of the Sn^2+^/Dop^−^ species in NaCl aqueous solutions.

*T*/K.	*I*/mol dm^−3^	logβ_M2L2_ ^a^	logβ_ML2_ ^a^	logβ_MLOH_ ^a^	logβ_M2LOH_ ^a^
288.15	0.150	35.21 ± 0.09 ^b^	24.51 ± 0.01 ^b^	9.54 ± 0.01 ^b^	15.43 ± 0.06 ^b^
298.15	0.147	34.83 ± 0.01	24.12 ± 0.01	9.49 ± 0.01	15.67 ± 0.03
298.15	0.472	34.35 ± 0.01	23.10 ± 0.01	9.10 ± 0.01	15.55 ± 0.02
298.15	0.709	33.95 ± 0.01	22.51 ± 0.01	8.74 ± 0.01	15.96 ± 0.01
298.15	0.954	31.68 ± 0.07	20.75 ± 0.03	7.63 ± 0.02	15.60 ± 0.05
310.15	0.150	32.54 ± 0.09	20.89 ± 0.02	8.47 ± 0.06	14.90 ± 0.01

^a^ Refers to the general equilibrium: pM^n+^ + qL^z−^ + rH^+^ = M_p_L_q_H_r_^(np−zq+r)^; ^b^ ±Std. Dev.

**Table 7:** Formation constants at infinite dilution and different temperatures of the CH_3_Hg^+^/Dop^−^ species.

	log^T^β_ML_ ^b^	log^T^β_MLH_ ^b^	log^T^β_MLCl_ ^b^	log^T^β_MLOH_ ^b^
	*T* = 288.15 K			
*I*→0 ^a^	10.80 ± 0.02 ^c^	19.52 ± 0.03 ^c^	11.44 ± 0.02 ^c^	1.99 ± 0.06 ^c^
*C* ^d^	1.58 ± 0.04	−0.40 ± 0.03	1.68 ± 0.04	−0.16 ± 0.03
	*T* = 298.15 K			
*I*→0	10.62 ± 0.04	19.65 ± 0.05	11.42 ± 0.02	2.00 ± 0.04
*C*	0.30 ± 0.02	2.18 ± 0.11	0.07 ± 0.06	0.99 ± 0.14
	*T* = 310.15 K			
*I*→0	10.48 ± 0.02	19.43 ± 0.01	11.16 ± 0.02	2.01 ± 0.03
*C*	−0.62 ± 0.02	0.49 ± 0.04	−0.49 ± 0.03	−0.24 ± 0.04
	*T* = 318.15 K			
*I*→0	9.79 ± 0.08	19.56 ± 0.02	11.50 ± 0.02	2.02 ± 0.02
*C*	−0.68 ± 0.10	−0.49 ± 0.03	−0.38 ± 0.02	−0.10 ± 0.01

^a^*I*/mol dm^−3^; ^b^ refers to the general equilibrium: pM^n+^ + qL^z−^ + rH^+^ + Cl^−^ = M_p_L_q_H_r_Cl^(np−zq+r−1)^; ^c^ ±Std. Dev.; ^d^ parameter for the dependence of logβ_pqr_ on *I*/mol dm^−3^.

**Table 8:** Formation constants at infinite dilution and different temperatures of the Mg^2+^/Dop^−^ species.

	log^T^β_ML_ ^b)^	log^T^β_MLOH_ ^b)^
	*T* = 288.15 K	
*I*→0 ^a)^	3.895 ± 0.040 ^c)^	−5.291 ± 0.090 ^c)^
*C* ^d)^	0.190 ± 0.072	−0.692 ± 0.144
	*T* = 298.15 K	
*I*→0	3.560 ± 0.036	−5.366 ± 0.108
*C*	0.140 ± 0.090	−0.612 ± 0.152
	*T* = 310.15 K	
*I*→0	3.007 ± 0.170	−4.796 ± 0.094
*C*	−0.007 ± 0.223	−1.239 ± 0.146

^a)^*I*/mol dm^−3^; ^b)^ refers to the general equilibrium: pM^n+^ + qL^z−^ + rH^+^ = M_p_L_q_H_r_^(np−zq+r)^; ^c)^ ±Std. Dev.; ^d)^ parameter for the dependence of logβ_pqr_ on *I*/mol dm^−3^.

**Table 9:** Formation constants at infinite dilution and *T* = 298.15 K for the M^n+^/Dop^−^ species and enthalpy and entropy change values.

Species	log^T^β_pqr_ ^a^	*C* ^b^	∆H ^c^	T∆S ^c^
		CH_3_Hg^+^/Dop^−^		
ML	10.62 ± 0.03 ^d^	0.27 ± 0.06 ^d^	−76 ± 5 ^d^	−15 ± 5 ^d^
MLH	19.64 ± 0.05	0.01 ± 0.10	−8 ± 5	104 ± 5
MLCl	11.41 ± 0.02	0.08 ± 0.05	−34 ± 2	31 ± 2
MLOH	2.00 ± 0.04	−0.04 ± 0.05	2 ± 3	13 ± 3
		Ca^2+^/Dop^−^		
ML	5.30 ± 0.07 ^d^	−0.88 ± 0.12 ^d^	-	
MLH	13.80 ± 0.06	0.33 ± 0.15	−58 ± 25 ^d,e^	20 ± 25 ^d,e^
MLOH	−6.18 ± 0.06	−0.06 ± 0.09	99 ± 43	64 ± 43
		Mg^2+^/Dop^−^		
ML	3.560 ± 0.036 ^d^	0.07 ± 0.04 ^d^	−72 ± 2	52 ± 2 ^d^
MLOH	−5.366 ± 0.108	−1.03 ± 0.03	24 ± 3	−7 ± 3
		Sn^2+^/Dop^−^		
M_2_L_2_	36.40 ± 0.29 ^d^	−2.90 ± 0.50 ^d^	−211 ± 28 ^d,e^	−4 ± 28 ^d,e^
ML_2_	25.56 ± 0.20	−3.33 ± 0.36	−286 ± 35	−141 ± 35
MLOH	10.43 ± 0.19	−1.75 ± 0.33	−84 ± 20	−24 ± 21
M_2_L(OH)	16.11 ± 0.04	0.30 ± 0.06	−42 ± 21	50 ± 21

^a^ Refers to the general equilibrium: pM^n+^ + qL^z−^ + rH^+^ + Cl^−^ = M_p_L_q_ClH_r_^(np−zq+r−1)^; ^b^ parameter for the dependence on *I*/mol dm^−3^; ^c^ enthalpy and entropy change values of formation in kJ/mol; ^d^ ±Std. Dev.; ^e^ calculated at *I* = 0.15 mol dm^−3^ and *T* = 298.15 K.

**Table 10:** Specific ion interaction parameters and Setschenow coefficients for the species of the metal/dopamine systems.

		CH_3_Hg^+^		
	**∆ε_ML_ ^a^**	**∆ε_MLH_ ^a^**	**∆ε_MLCl_ ^a^**	**∆ε_MLOH_ ^a^**
*T* = 288.15 K	1.53 ± 0.02 ^b^	−0.88 ± 0.06	1.61 ± 0.02	−0.15 ± 0.03
*T* = 298.15 K	0.38 ± 0.02	2.43 ± 0.15	0.48 ± 0.04	0.99 ± 0.15
*T* = 310.15 K	−0.64 ± 0.02	0.31 ± 0.04	−0.52 ± 0.02	−0.24 ± 0.03
		Ca^2+^		
*T* = 298.15 K	−1.11 ± 0.07	0.19 ± 0.09	-	−0.16 ± 0.06
		Mg^2+^		
*T* = 288.15 K	0.26 ± 0.06	-	-	−1.22 ± 0.04
*T* = 298.15 K	0.06 ± 0.07	-	-	−1.02 ± 0.06
*T* = 310.15 K	0.11 ± 0.06	-	-	−1.63 ± 0.02
		Sn^2+^		
	**∆ε_M2L2_^a^**	**∆ε_ML2_ ^a^**	**∆ε_MLOH_ ^a^**	**∆ε_M2LOH_ ^a^**
*T* = 298.15 K	−2.88 ± 0.19 ^b^	−3.29 ± 0.14	−1.72 ± 0.13	0.30 ± 0.02
Species		Ca^2+^	Mg^2+^	Sn^2+^
*T* = 298.15 K				
ε_(ML+,Cl^−^)_ ^c^		1.03 ± 0.07 ^b^	−0.20 ± 0.07	-
ε_(MLH2+,Cl^−^)_ ^c^		−0.16 ± 0.09	-	-
*k*_MLOH_ ^c,d^		−0.06 ± 0.01	0.99 ± 0.05	1.39 ± 0.13
ε_(M2L22+,Cl^−^)_ ^c^		-	-	2.49 ± 0.19
*k*_ML2_ ^c,d^		-	-	2.90 ± 0.14
ε_(M2LOH2+,Cl^−^)_ ^c^		-	-	−0.60 ± 0.02

^a^ Calculated by means of Equation (10); ^b^ ±Std. Dev.; ^c^ Calculated by means of Equations (12)–(15); ^d^ Setschenow coefficient for the neutral species calculated by means of Equation (11).

**Table 11:** Sequestering ability of dopamine towards the metal ions at *I* = 0.15 mol dm^−3^, pH = 7.4 and different temperatures.

Metal	*T*/K	pL_0.5_
	288.15	2.36
CH_3_Hg^+^	298.15	2.63
	310.15	3.74
	318.15	4.50
Mg^2+^	288.15	2.23 ^a)^
	298.15	2.27 ^a)^
	310.15	2.94 ^a)^
	288.15	0.81
Ca^2+^	298.15	1.15
	310.15	1.86
	288.15	3.74
Sn^2+^	298.15	3.46
	310.15	3.75

^a)^ Owing to the low values at pH = 7.4, for Mg^2+^ the pL_0.5_ were calculated at pH = 9.5.

**Table 12:** Sequestering ability of dopamine towards metal ions at *T* = 298.15 K, pH = 7.4 and different ionic strengths.

Metal	*I*/mol dm^−3^	pL_0.5_
	0.15	2.63
CH_3_Hg^+^	0.50	2.90
	0.75	3.02
	1.00	3.12
	0.15	2.27 ^a^
Mg^2+^	0.50	2.11 ^a^
	0.75	2.04 ^a^
	1.00	2.10 ^a^
	0.15	1.15
Ca^2+^	0.50	1.19
	0.75	1.68
	1.00	2.58
	0.15	3.74
Sn^2+^	0.50	3.70
	0.75	3.12
	1.00	2.60

^a^ Calculated at pH = 9.5.

**Table 13:** Sequestering ability of dopamine towards metal ions at *I* = 0.15 mol dm^−3^, *T* = 298.15 K and different pHs.

			pL_0.5_		
**pH**	CH_3_Hg^+^	CH_3_Hg^+ a^	Ca^2+^	Mg^2+^	Sn^2+^
5	-	-	-	-	1.30
6	-	-	-	-	2.33
7.4	2.63	3.74	1.15	0.08	3.74
8.2	3.46	4.40	2.00	1.06	4.31
9	4.51	5.18	3.03	1.31	5.07
9.5	5.23	5.75	3.65	2.27	-
10.5	6.16	6.16	4.72	3.97	-

^a^ Calculated at *T* = 310.15 K.

**Table 14:** Empirical parameters for the modeling of pL_0.5_ values of the metal/dopamine systems with respect to *T*/K, *I*/mol dm^−3^ and pH.

Metal	p_1_	p_2_	p_3_	p_4_	Std. Dev. ^b^	Mean Dev. ^b^
CH_3_Hg^+^	−19.0 ± 2.6 ^a^	0.050 ± 0.007	−0.08 ± 0.20	0.97 ± 0.03	0.14	0.27
Mg^2+^	−18.8 ± 4.0	0.035 ± 0.013	−0.37 ± 0.19	1.12 ± 0.09	0.12	0.24
Ca^2+^	−22.4 ± 2.6	0.050 ± 0.009	1.33 ± 0.12	1.16 ± 0.02	0.05	0.09
Sn^2+^	−3.8 ± 2.0	0.001 ± 0.007	−1.04 ± 0.08	0.95 ± 0.03	0.07	0.10

^a^ ±Std. Dev.; ^b^ standard and mean deviation on the fit of Equation (20).

**Table 15:** Thermal decomposition steps for the Ca^2+^/Dop^−^ precipitates.

Scheme.	Percentages by Weight at the Beginning of the Process	Percentage Loss of Weight during the Decomposition Process
*Ca-Dop(A)*		
1° process	99.8%	9.45%
2° process	90.31%	34.49%
3° process	55.82%	17.96%
4° process	37.82%	3.10%
5° process	34.77%	10.56%
Stoichiometry (M:L)	1:1	
*Ca-Dop(B)*		
1° process	99.76%	14.77%
2° process	84.98%	57.02%
3° process	27.97%	15.22%
4° process	12.75%	3.89%
Stoichiometry (M:L)	1:3	
*Ca-Dop(C)*		
1° process	99.86%	10.01%
2° process	89.85%	67.93%
3° process	21.92%	11.79%
4° process	10.12%	3.48%
Stoichiometry (M:L)	1:5	

**Table 16:** Literature data for the complexation of dopamine towards metal ions.

*I*/mol dm^−3^	*T*/K	Metal	Medium	logβ_ML_	logβ_MLH_	logβ_ML2_	logβ_M(LH)2_	logβ_ML2H_		Ref.
0.1	298.15	Cu^2+^	HClO_4_	20.56	26.91		48.26			[43]
0.2	298.15	Y^3+^	KCl	7.95		14.84				[44]
		La^3+^		6.35		11.7				[45]
0.2	288.15	Mg^2+^	NaCl	4.57						[42]
	298.15			4.50						
	308.15			4.49						
0.2	288.15	Zn^2+^	NaCl				52.94			[46]
	298.15						52.14			
	308.15						51.22			
0.15	310.15	Cu^2+^	NaClO_4_	15.44	22.07	23.25				[47]
		Ni^2+^		9.66	18.12	26.07				
		Zn^2+^		11.43	18.89					
0.2	288.15	MoO_4_^2−^	NaCl				51.48			[48]
	298.15						51.08			
	308.15						50.66			
0.2	298.15	Cu^2+^	KCl	16.60	24.22	24.78	45.83	35.66		[49]
		Ni^2+^		9.42	19.38	14.81	35.66	25.61		
		Zn^2+^			20.21	18.05	38.93	28.67		
0.37	293.15	Ni^2+^	NaNO_3_		18.373	13.86	34.05			[50]
		Cu^2+^		16.01	23.29	23.47	44.26			
		Zn^2+^			19.33					
		Cd^2+^			17.991					
		Pb^2+^			22.23					

## Supplementary Materials

The following are available online at https://www.mdpi.com/article/10.3390/biom11091312/s1, Table S1. Literature protonation constants of dopamine at different ionic strengths and temperatures in NaCl aqueous solutions; Table S2. Literature hydrolytic and chloride constants of CH_3_Hg^+^, Mg^2+^, Ca^2+^ and Sn^2+^ ions in NaCl aqueous solutions at different ionic strengths and temperatures; Table S3. Formation constants of the CH_3_Hg^+^/Dop^−^ complexes at different ionic strengths and temperatures in molal concentration scale; Table S4. Formation constants of the Ca^2+^/Dop^−^ complexes at different ionic strengths and temperatures in molal concentration scale; Table S5. Formation constants of the Mg^2+^/Dop^−^ complexes at different ionic strengths and temperatures in molal concentration scale; Table S6. Formation constants of the Sn^2+^/Dop^−^ complexes at different ionic strengths and temperatures in molal concentration scale; Figure S1. Distribution diagram for the Mg^2+^/Dop^−^ species at *I* = 0.15 mol dm^−3^ and *T* = 310.15 K; Figure S2. Derivative curves for the different Ca^2+^/Dop^−^ precipitates; Figure S3. Thermogravimetric profile of the percentage weight loss for Ca^2+^/Dop^−^ A; Figure S4. Thermogravimetric profile of the percentage weight loss for Ca^2+^/Dop^−^ C.
