# Supplementary material for: The Solution Behavior of Dopamine in the Presence of Mono and Divalent Cations: A Thermodynamic Investigation in Different Experimental Conditions
Source: Biomolecules. 2021 Sep 5;11(9):1312. doi: 10.3390/biom11091312 (PMC8466117; doi:10.3390/biom11091312)
Supplement: Supplementary file 1 [file biomolecules-11-01312-s001.zip › biomolecules-1345734-supplementary.pdf]

biomolecules

Supplementary Materials

Table S1. Literature protonation constants of dopamine at different ionic strengths and temperatures in NaCl aqueous solutions.

I/mol dm^{-3}	log K_1^{H} [a]	I/ mol dm^{-3}	log K_2^{H} [a]	I/ mol dm^{-3}	log K_1^{H} [a]	I/ mol dm^{-3}	log K_2^{H} [a]
		T = 288.15 K				T = 298.15 K	
0.147	10.687	0.147	9.496	0.162	10.566	0.162	9.209
0.491	10.743	0.491	8.923	0.197	10.573	0.197	8.963
0.747	10.941	0.747	8.951	0.491	10.440	0.491	9.066
0.982	11.029	0.982	9.018	0.504	10.399	0.511	9.119
				0.735	10.307	0.735	8.971
				0.980	10.262	0.980	8.878
		T = 310.15 K				T = 318.15 K	
0.148	9.989	0.148	8.376	0.148	9.519	0.148	8.144
0.165	9.988	0.165	8.690	0.493	9.136	0.493	8.257
0.495	9.904	0.497	8.470	0.749	9.213	0.749	8.307
0.743	9.978	0.746	8.514	0.986	8.798	0.986	8.312
0.989	10.002	0.985	8.513				

[a] Refers to the general equilibrium $H_{i-1}L^{(z+i-1)} + H^{+} = H_iL^{-z+i}$

Table S2. Literature hydrolytic and chloride constants of CH_3Hg^+, Mg^{2+}, Ca^{2+} and Sn^{2+} ions in NaCl aqueous solutions at different ionic strengths and temperatures.

Species		CH_3Hg^+			
		T/K			
	I/ mol dm^{-3}	288.15 K	298.15 K	310.15 K	318.15 K
		log β_{pr}			
CH_3HgOH^0	0.15	-4.52	-4.54	-4.50	-4.50
	0.50	-4.48	-4.58	-4.46	-4.46
	0.75	-4.45	-4.64	-4.44	-4.43
	1.00	-4.42	-4.64	-4.41	-4.40
CH_3HgCl^0	0.15	5.33	5.25	5.34	5.33
	0.50	5.31	5.16	5.31	5.30
	0.75	5.32	5.13	5.32	5.31
	1.00	5.34	5.13	5.34	5.33
Species		Mg^{2+}			
$MgOH^+$	0.15	-11.21	-11.22	-11.22	
	0.50	-11.22	-11.23	-11.23	
	0.75	-11.27	-11.28	-11.28	
	1.00	-11.33	-11.33	-11.34	
$Mg_4(OH)_4^+$	0.15	-37.46	-37.46	-37.47	
	0.50	-37.83	-37.85	-37.88	
	0.75	-37.97	-38.00	-38.04	
	1.00	-38.09	-38.13	-38.18	
Species		Ca^{2+}			
	0.15	-13.14	-12.98	-12.56	
$CaOH^+$	0.50		-12.98		
	0.75		-12.98		
	1.00		-12.97		

Species	Sn^{2+}					
T/K	288.15	298.15				310.15
I/mol dm^{-3}	0.15	0.15	0.50	0.75	1.00	0.15
$SnOH^+$	3.96	-3.78	-3.91	-3.96	-4.00	-3.56
$Sn(OH)_2^0$	6.67	-6.53	-6.68	-6.75	-6.81	-6.36
$Sn(OH)^{3-}$	6.53	-16.97	-17.05	-17.1	-17.15	-17.5
$Sn2(OH)_2^{2-}$	5.36	-5.06	-5.2	-5.26	-5.32	-4.7
$Sn_3(OH)_4^{2+}$	7.00	-6.39	-6.63	-6.72	-6.8	-5.66
$SnCl^+$	0.7	0.76	0.58	0.52	0.49	0.77
$SnCl_2^0$(aq)	1.44	1.5	1.23	1.15	1.09	1.76
$SnCl_3^-$	1.38	1.46	1.19	1.11	1.05	1.71
$SnCl(OH)^0$(aq)	2.11	-2.07	-2.36	-2.5	-2.61	-2.98

[a] Refers to the general equilibrium: $p\ M^{n+} + r\ H_2O = M_p(OH)_r^{(np-r)} + r\ H^+$

Table S3 Formation constants of the CH_3Hg^+/Dop^- complexes at different ionic strengths and temperatures in molal concentration scale.

T/K	*I*/mol kg^{-1}	log β_{ML} [a)]	log β_{MLOH} [a)]
288.15	0.000	3.895	-5.291
288.15	0.151	3.458	-6.278
288.15	0.505	3.255	-6.676
288.15	0.760	3.261	-6.905
288.15	1.019	3.459	-7.336
298.15	0.000	3.559	-5.366
298.15	0.151	3.032	-6.111
298.15	0.506	2.792	-6.531
298.15	0.763	2.770	-6.825
298.15	1.022	2.942	-7.324
310.15	0.000	3.004	-4.796
310.15	0.151	2.557	-5.926
310.15	0.508	2.274	-6.369
310.15	0.766	2.223	-6.736
310.15	1.027	2.364	-7.311

a) Refers to the general equilibrium: p M^{n+} + q L^{z-} + r H^+ + Cl^- = $M_pL_qH_rCl^{(np-zq+r-1)}$

Table S4 Formation constants of the Ca^{2+}/Dop^- complexes at different ionic strengths and temperatures in molal concentration scale.

T/K	*I*/mol Kg^{-1}	logβ_{ML} [a)]	logβ_{MLH} [a)]	logβ_{MLOH} [a)]
**288.15**	0.150	-	*13.60*	*-6.97*
**298.15**	0.145	4.82	13.42	-5.78
**298.15**	0.483	3.64	13.26	-7.78
**298.15**	0.714	23.94	13.62	-6.69
**298.15**	0.972	5.24	14.40	-6.49
**310.15**	0.150	-	*13.50*	*-5.60*

a) Refers to the general equilibrium: p M^{n+} + q L^{z-} + r H^+ = $M_pL_qH_r^{(np-zq+r)}$

Table S5 Formation constants of the Mg^{2+}/Dop^- complexes at different ionic strengths and temperatures in molal concentration scale.

T/K	*I*/mol Kg^{-1}	$\log\beta_{ML}$ a)	$\log\beta_{MLOH}$ a)
288.15	0.000	3.89	-5.29
288.15	0.151	3.46	-6.28
288.15	0.505	3.25	-6.68
288.15	0.760	3.26	-6.91
288.15	1.019	3.46	-7.34
298.15	0.000	3.56	-5.37
298.15	0.151	3.03	-6.11
298.15	0.506	2.79	-6.53
298.15	0.763	2.77	-6.83
298.15	1.022	2.94	-7.32
310.15	0.000	3.00	-4.80
310.15	0.151	2.56	-5.93
310.15	0.508	2.27	-6.37
310.15	0.766	2.22	-6.74
310.15	1.027	2.36	-7.31

a) Refers to the general equilibrium: $p\ M^{n+} + q\ L^{z-} + r\ H^+ = M_pL_qH_r^{(np-zq+r)}$

Table S6 Formation constants of the Sn^{2+}/Dop^- complexes at different ionic strengths and temperatures in molal concentration scale.

***T*/K**	***I*/mol Kg^{-1}**	**$\log\beta_{M2L2}$ a)**	**$\log\beta_{ML2}$ a)**	**$\log\beta_{MLOH}$ a)**	**$\log\beta_{M2LOH}$ a)**
**288.15**	0.151	35.20	24.50	9.53	15.42
**298.15**	0.148	34.82	24.11	9.49	15.67
**298.15**	0.477	34.33	23.09	9.1	15.54
**298.15**	0.720	33.93	22.50	8.74	15.95
**298.15**	0.974	31.65	20.73	7.63	15.59
**310.15**	0.151	32.53	20.88	8.46	14.89

a) Refers to the general equilibrium: $p\ M^{n+} + q\ L^{z-} + r\ H^+ = M_pL_qH_r^{(np-zq+r)}$

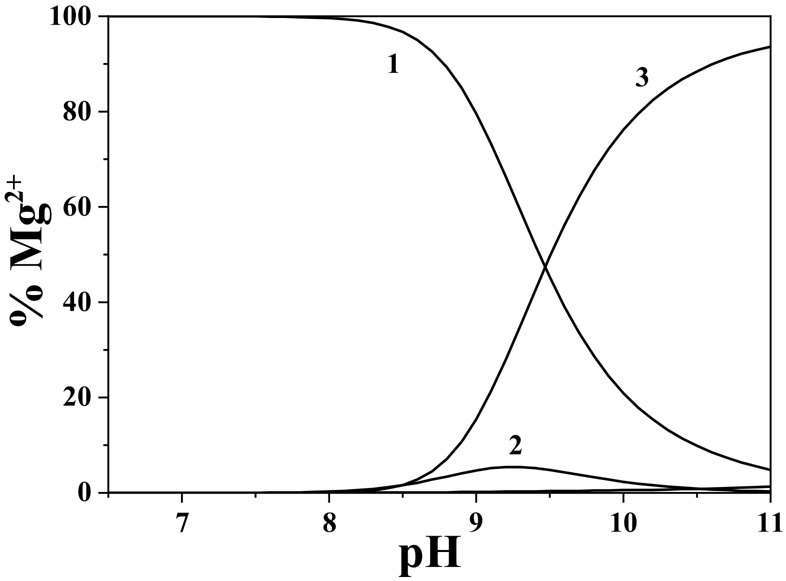

Figure S1. Distribution diagram for the Mg^{2+}/Dop^{-} species at I = 0.15 mol dm^{-3} and T = 310.15 K. Species: 1 M, 2 ML, 3 MLOH. (Experimental Conditions: $c_{Mg^{2+}}$ = 1.0 mmol dm^{-3}; $c_{DOP^{-}}$ = 1.0 mmol dm^{-3}) [M = Mg^{2+}; L = Dop^{-}].

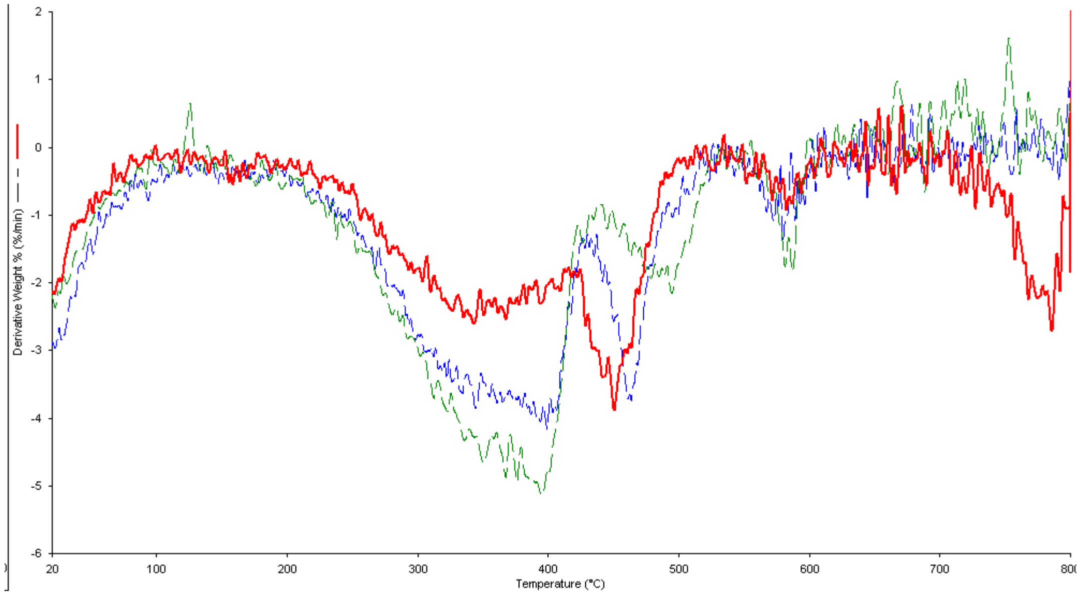

Figure S2. Derivative weight curves for the different Ca^{2+}/Dop^{-} precipitates.

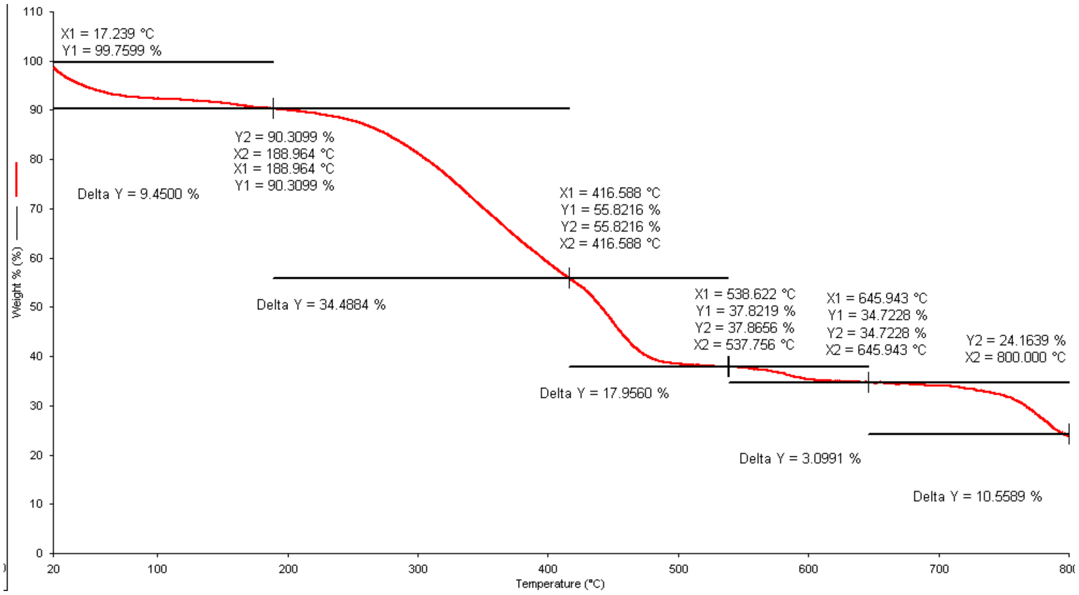

Figure S3. Thermogravimetric profile of the percentage weight loss for Ca^{2+}/Dop^{-} A.

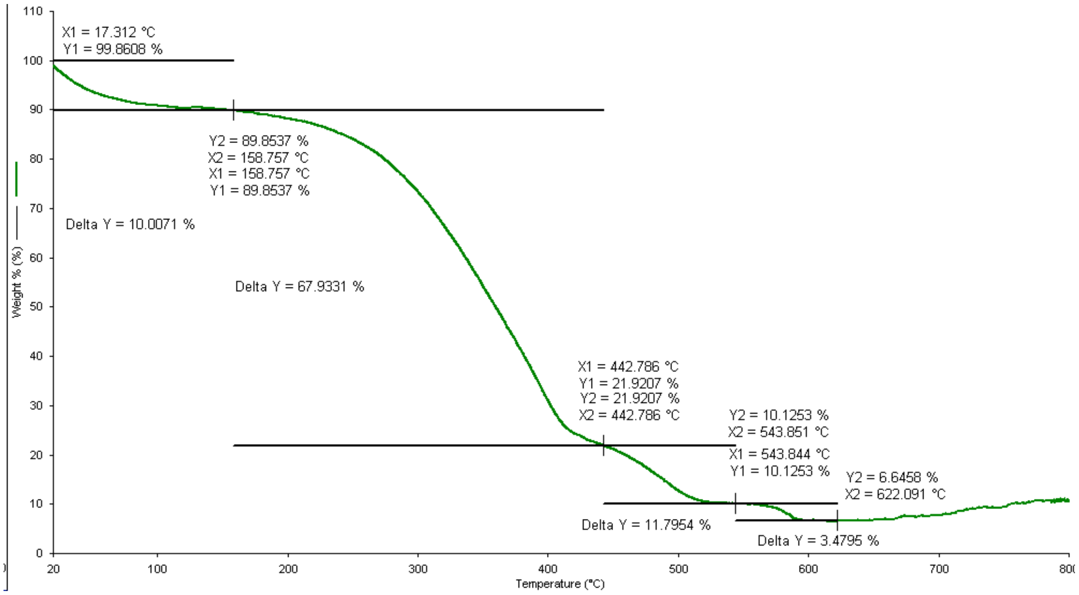

Figure S4. Thermogravimetric profile of the percentage weight loss for Ca^{2+}/Dop^{-} C.
